# Oral Microbiota Profile Associates with Sugar Intake and Taste Preference Genes

**DOI:** 10.3390/nu12030681

**Published:** 2020-03-03

**Authors:** Anders Esberg, Simon Haworth, Pamela Hasslöf, Pernilla Lif Holgerson, Ingegerd Johansson

**Affiliations:** 1 Department of Odontology/Section of Cariology, Umeå University, 901 87 Umeå, Sweden; ingegerd.johansson@umu.se; 2 Medical Research Council Integrative Epidemiology Unit, Department of Population Health Sciences Bristol Medical School, University of Bristol, Bristol BS8 2BN, UK; simon.haworth@bristol.ac.uk; 3 Bristol Dental School, University of Bristol, Bristol BS1 2LY, UK; 4 Department of Odontology/Section of Pediatric Dentistry, Umeå University, 901 87 Umeå, Sweden; pamela.hasslof@umu.se (P.H.); pernilla.lif@umu.se (P.L.H.)

**Keywords:** microbiota, saliva, sugar, taste, genes, 16S rDNA sequencing

## Abstract

Oral microbiota ecology is influenced by environmental and host conditions, but few studies have evaluated associations between untargeted measures of the entire oral microbiome and potentially relevant environmental and host factors. This study aimed to identify salivary microbiota cluster groups using hierarchical cluster analyses (Wards method) based on 16S rRNA gene amplicon sequencing, and identify lifestyle and host factors which were associated with these groups. Group members (*n* = 175) were distinctly separated by microbiota profiles and differed in reported sucrose intake and allelic variation in the taste-preference-associated genes *TAS1R1* (rs731024) and *GNAT3* (rs2074673). Groups with higher sucrose intake were either characterized by a wide panel of species or phylotypes with fewer aciduric species, or by a narrower profile that included documented aciduric- and caries-associated species. The inferred functional profiles of the latter type were dominated by metabolic pathways associated with the carbohydrate metabolism with enrichment of glycosidase functions. In conclusion, this study supported in vivo associations between sugar intake and oral microbiota ecology, but it also found evidence for a variable microbiota response to sugar, highlighting the importance of modifying host factors and microbes beyond the commonly targeted acidogenic and acid-tolerant species. The results should be confirmed under controlled settings with comprehensive phenotypic and genotypic data.

## 1. Introduction

The gastro-intestinal canal is heavily colonized, with over 700 bacterial species or unnamed phylotypes in the oral cavity alone [1]. The bacteria form niche-specific ecosystems with patterns of bacterial cohabitation due to influences from factors like transmission, host receptor availability, pH, access of nutrients, oxygen and symbiosis or communication with nearby species [2,3]. Generally, these ecosystems are in equilibrium but shifts may occur due to medical or lifestyle changes [4,5,6].

Many studies report associations between host factors, including saliva quality, pH regulation, and genetics, and single or panels of bacteria [7,8,9], but few studies have evaluated the associations between host traits and the oral microbiota in an untargeted manner [10,11,12]. Carbohydrate, especially high sugar, intake is reported to correlate with the enrichment of acidogenic and acid-tolerant caries-associated species. As an example, frequent glucose pulses (low *p*H) on a tooth-mimicking biofilm with nine bacterial species enumerated species that thrive at a low *p*H, whereas others were relatively reduced [13]. This shift mirrors the tooth biofilm dysbiosis in dental caries [14]. Sucrose exposure in vivo and assessments of targeted species support these experimental findings [15,16,17]. However, associations between sugar intake and untargeted characterization of the oral microbiota remain sparsely studied [4]. That genetic variation plays a fundamental role in individual differences in food preference and thereby food selection has been described from studies targeting candidate genes and in genome wide studies (GWAS) as comprehensively reviewed [18,19]. However, non-genetic and non-lifestyle linked factors have also been indicated to influence food habits, i.e., oral microbiota as well as gut microbiota have been suggested to modulate taste perception and eating behaviours [20,21,22,23]. We, and others, have shown that the intake of and preference for sweet foods are associated with polymorphisms in sweet and bitter taste receptor encoding genes, such as *TAS1R2, TAS1R3* and *TAS2R38,* but also glucose transporter genes (*SCL2* and *SCL4*) and the gustducin-encoding (*GNAT3*) gene [24,25].

The aim of the study was to explore the global saliva microbiota structure, identify groups of subjects defined by similar oral microbiota profiles in Swedish young adults, and search for potentially associated lifestyle and host factors.

## 2. Materials and Methods

### 2.1. Study Subjects

Teenagers and young adults attending one public dental health care clinic in the city of Umeå, Sweden were recruited consecutively as they attended the dentist’s office for their regular dental health control. Those who had received antibiotic treatment in the preceding 6 months, had a systemic disease or were taking regular medication, or were unable to communicate in Swedish or English, were not approached. In total, 176 participants in the age range 17–21 years were eligible and consented to participate.

The project received ethical approval by the Swedish Ethical Review Authority (Dnr 2012-111-31M) with an addendum (Dnr 2015-389-32M), and it adhered to the Helsinki Declaration and the General Data Protection Regulation (GDPR). The project is reported in accordance with Strengthening the Reporting of Observational Studies in Epidemiology (STROBE) guidelines for cohort studies.

### 2.2. Saliva Sampling, Bacteria Culturing and DNA Extraction

Approximately 3 mL of chewing stimulated saliva was collected into ice-chilled test tubes. Colony-forming units (CFUs) of mutans streptococci (*Streptococcus mutans* and *Streptococcus sobrinus*) and lactobacilli were assessed per ml of fresh saliva by cultivation of 100 μL on mitis salivarius sucrose agar supplemented with 0.2 U of bacitracin (MSB) and lactobacillus selective (LBS) agar, respectively. The plates were incubated at 37°C in 5% CO_2_ for 48 h. The remaining saliva was stored at −80°C.

Genomic DNA was extracted from saliva samples, positive and negative controls using the GenElute™ Bacterial Genomic DNA Kit (Sigma-Aldrich, St. Louis, MO, USA). Briefly, the samples were centrifuged for 5 min at 13,000 rpm, lysed in buffer with lysozyme and mutanolysin, treated with RNase and Proteinase K, and purified and washed. All DNA extractions were done at the laboratory at the Dental School, Umeå University, Sweden by the same person and with kits from the same batch. The quality of the extracted DNA was estimated using NanoDrop 1000 Spectrophotometer (Thermo Fisher Scientific, Uppsala, Sweden) and the quantity by the Qubit 4 Fluorometer (Invitrogen, Thermo Fisher Scientific, Waltham, MA, USA). The mean yield from 200 μL saliva was 32 ng/μL (range 6–92 ng/μL) and the ratio of the absorbances at 260 and 280 nm was 1.8 or higher. Mixtures of known mock bacteria were used as positive controls and sterile water as negative control.

### 2.3. 16S rRNA Gene Amplicon Generation and Sequencing

Bacterial 16S rRNA gene amplicons were generated from the variable regions v1-v3 (27F-forward AGAGTTTGATCATGGCTCAG and 530R-reverse GTATTACCGCGGCTGCTG primers), and v3-v4 (357F-forward TACGGGAGGCAGCAG and 800R-reverse CCAGGGTATCTAATCC primers) from saliva and a mock community DNA. Library preparation, sequencing on the Illumina Miseq platform, and sequence demultiplexing were done at Eurofins Genomics (Ebersberg, Germany) according to their standard protocols. The company provided demultiplexed FASTQ-files, which were imported to QIIME2 [26], and DADA2 was used for denoising, pair end read fusion, chimeric sequence removal [27], and the identification of 100% identical amplicon sequence variants (ASVs) per sample [28]. Default parameters in DADA2 were used with left trimming of 13 bp for both forward and reversed reads, right trimming at 230 bp for the reversed sequences and 268 bp for the forward sequences. ASVs with >1 read were blasted against the extended Human Oral Microbiome Database (eHOMD, http://www.homd.org) for taxonomic annotation [29]. In the eHOMD blast, only ASVs with at least 98.5% identity with a named species or unnamed phylotype in eHOMD were retained, and those with the same HMT number were aggregated. The negative control contained <50 sequences and the positive control mock species were correct for representative sequences with 25 or more reads. Therefore, all comparisons were based on taxa with at least 25 reads. For simplicity, all taxa are referred to as species in the text. Sequencing failed for one saliva sample, leaving 175 samples in the final analyses.

### 2.4. Diet Recording

The participants reported their diet intake in a food frequency questionnaire (FFQ, http://www.matval.se). The FFQ is a semi-quantitative questionnaire, with questions on 93 food items/food aggregates selected to represent the habitual intake in Sweden and includes questions on alcoholic beverages. Participants were asked to report their typical intake in the last year. Intakes were reported on an increasing, nine-level scale, from never to four or more times a day. Portion sizes were estimated from photographs showing four portion sizes of staple foods (potatoes, rice, and pasta), meat/fish and vegetables, or standard food weights, such as for an egg or apple. Sucrose intake was estimated from nine questions, i.e., (i) fruit soup with or without a thickening agent, (ii) buns and biscuits, (iii) cookies and cakes, (iv) marmalade and jam, (v) ice cream, (vi) sodas, (vii) syrups, (viii) sugar, (ix) sweets including candies and chocolate. For sugar, the sum of mono and disaccharides (excluding lactose) from fruits, berries, vegetables, juices and honey was added. The FFQ included one question on how often the respondent ate or drank sweet products without sugar, i.e., with a sugar substitute.

Intake of energy and energy-providing nutrients was calculated by multiplying intake frequencies by portion sizes and weighting by the energy/nutrient contents in the food composition database at the National Food Administration (https://www.livsmedelsverket.se/en/food-and-content/naringsamnen).

A Healthy Diet Score that reflects healthy eating habits was calculated as previously described [30]. Briefly, frequency of intake per day was calculated for eight food/beverage groups. Favourable food groups included fish, fruits (except juices), vegetables (except potatoes) and whole grains. Unfavourable food/beverage groups included red or processed meats, desserts and sweets, sugar-sweetened beverages and fried potatoes. Intake frequencies were ranked within each sex in ascending quartile ranks for favourable foods/beverage groups, and in descending quartile ranks for unfavourable foods/beverage groups. The sum of all quartile ranks represents the Healthy Diet Score, with a minimum of zero and a maximum of 24, and with higher ranks indicating healthier food and beverage choices.

The relative validity of FFQ-derived intakes has been estimated against 24 h dietary records and/or biological markers [31,32,33,34]. For sucrose reliability, measured as correlations between registrations done one year apart, results were 0.80 for men and 0.75 for women, and the relative validity against 10 repeated 24 h recalls were 0.69 and 0.62 for sweet foods for men and women, respectively [31].

To reduce potential recording bias, energy-providing nutrients were expressed as energy standardized values (E%) and 11 participants with unrealistic reported energy intakes were excluded from analyses involving diet assessments. This was based on food intake level (FIL) scores calculated to estimate energy intake relative to minimal energy needs [35].

### 2.5. Recording of Medical and Other Lifestyle Conditions

Information on health status, oral hygiene, tobacco use, alcohol intake and most recent antibiotic exposure was obtained from a questionnaire. Dental caries was scored from visual and radiographic examinations in the dentist’s office with optimal lightning. Tooth surfaces that were sound according to ICDAS [36], score 0, or had caries in the enamel (e) according to ICDAS scores =1 and 2, or had caries in the enamel with a localized breakdown with or without dentine involvement (D) according to ICDAS score ≥3, or with a filling (F), were recorded. The total numbers of decayed and filled tooth surfaces (DeFS) were calculated. The M component was not considered because tooth loss occurred for orthodontic reasons or severe hypomineralization in this study group.

### 2.6. Genotyping of Single Nucleotide Polymorphism in Taste Associated Genes

Genotyping of 121 Single Nucleotide Polymorphisms (SNPs) in the *TAS1R1*, *TAS1R2*, *TAS1R3*, *TAS2R16*, *TAS2R38*, *TAS2R50*, *SLC2A2*, *SLC2A4*, *GNAT3*, *CA6*, *SCN1B* and *TRPV1* taste-associated genes was performed at SciLife, Uppsala as described previously [24]. One SNP marker received a call rate of 0%. None of the remaining 120 SNPs deviated from Hardy–Weinberg equilibrium (*p* > 0.001), and they had an average call rate per sample of 99.8% and overall call rate of 99.8%. Genotyping data are uploaded at figshare (https://figshare.com/s/e292568e15c601e67a03).

### 2.7. Prediction of Functional Potential from the 16S rRNA Gene Information

Obtained representative ASVs were used to search for the potential molecular functions of the saliva microbiome using the 16S rRNA gene as marker gene, Phylogenetic Investigation of Communities by Reconstruction of Unobserved States (PICRUSt2) [37] and the Molecular Functions by orthology annotation (Kyoto Encyclopedia of Genes and Genomes (KEGG) orthology database, KO, https://www.genome.jp/kegg/kegg1.html) [38,39]. The steps included (i) creating a closed reference feature table in QIIME2 using the trained Greengenes dataset gg-13-8-99-nb-classifier.qza (Greengenes http://greengenes.lbl.gov, [40], (ii) qiime diversity core-metrics analysis in QIIME2, and (iii) export of pathway abundances and the feature table for down-stream analyses in KO and multivariate modelling. Group separation was tested by Euclidean distances in permutational multivariate analysis of variance (PERMANOVA), Bonferroni-corrected p-values and 9999 permutations. Follow-up functional enrichment analyses were done using the STRING database (version 10.5, https://string-db.org/) [41]. The same procedure was also done for eHOMD-defined species.

### 2.8. Data Handling and Statistical Analyses

Unsupervised hierarchical clustering (Ward’s method) was used to classify the 175 participants by the presence (or not) of ASVs and presence (or not) of species from eHOMD identification. The number of ASVs were standardized to the level of the sample with the fewest reads after DADA2 filtering (38,293 reads), and lowest per-sample abundance of eHOMD taxa, and transformed by inverse hyperbolic sine transformation, which defines log values, including for zero-values, which are prevalent for many ASVs and some species.

Continuous phenotypic variables were presented as means with 95% confidence interval limits (CI), and when adjusted for sex, age and body mass index (BMI) using generalized linear modelling. Differences were tested with non-parametric tests. For discrete measures, the percentages in groups were estimated and proportion differences tested with Chi^2^ test. SPSS version 25 (IBM Corporation, Armonk, NY, USA) was used for these analyses. All tests were controlled by the Benjamini and Hochberg procedure, and those with *p*-values <0.05 yielding an FDR of 5% are presented.

Alpha- and beta-diversities with associated PERMANOVA tests and ASV proportions for bar charts were calculated in QIIME 2.

Multivariate modelling was performed by partial least-square regressions (PLS) (SIMCA P+ version 15.0, Sartrius Stedim Data Analytics AB, Malmö, Sweden). PLS identifies directions in an X-swarm that characterize X well and are related to Y. The software scales all variables to unit variance, and performs a K-fold cross-validation where 1/7^th^ of the data are systematically kept out to fit a model and predict it from the remaining data (Q^2^-values). The results are displayed in scatter plots illustrating the separation of observations, and loading column plots displaying the mean correlation coefficient, with 95% CI between each predictor and the outcome variable. CIs that do not include zero are considered statistically significant.

The linear discriminant analysis effect size (LEfSe) method [42] was used to identify taxa effect size. Species that were shared between groups were identified in a Venn diagram [43].

## 3. Results

### 3.1. Study Group

**Figure 1:**
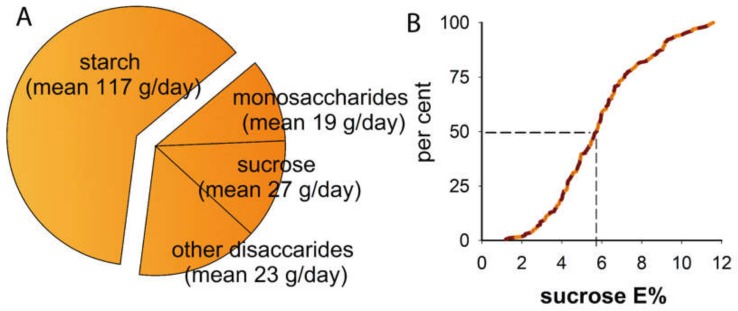
Total carbohydrate and sucrose intake in the study group. Proportions of various types of carbohydrates in the reported diet (**A**) and cumulative percentages for sucrose intake (**B**).

The study included a similar number of males and females. The mean BMI was in the normal range but with variation, and approximately one in five participants had a BMI >25. Sex-adjusted mean energy intake (after exclusion of the 11 participants with unrealistic energy intake) was 1856 kcal/day, with 40.3% coming from carbohydrates (E% COH) (Table 1). Starch was the dominant type of dietary carbohydrate (117 g/day) followed by sucrose (27 g/day), monosaccharides (19 g/day), and other sugars (23 g/day) (Figure 1A). Reported median intake of sucrose corresponded to 5.8% of the total energy intake (Figure 1B).

### 3.2. Overall Microbiota Assessment

For the 175 saliva samples, 14,317,039 sequences passed denoising and the removal of potential chimera with 1,685,662 reads referring to the v1–v3 and 12,631,377 sequences to the v3–v4 section. Under the present conditions, the v3–v4 sequencing yielded significantly more sequences per sample, better recognition of the mock species and higher diversity (Table S1). Further analyses were based on v3–v4 sequences.

The retained 12,631,377 v3–v4 sequences corresponded to 6171 representative amplicon sequence variants (ASVs) with ≥2 reads. These were in 13 phyla and 127 genera.

### 3.3. Cluster Classifications Based on ASV Pattern

**Figure 2:**
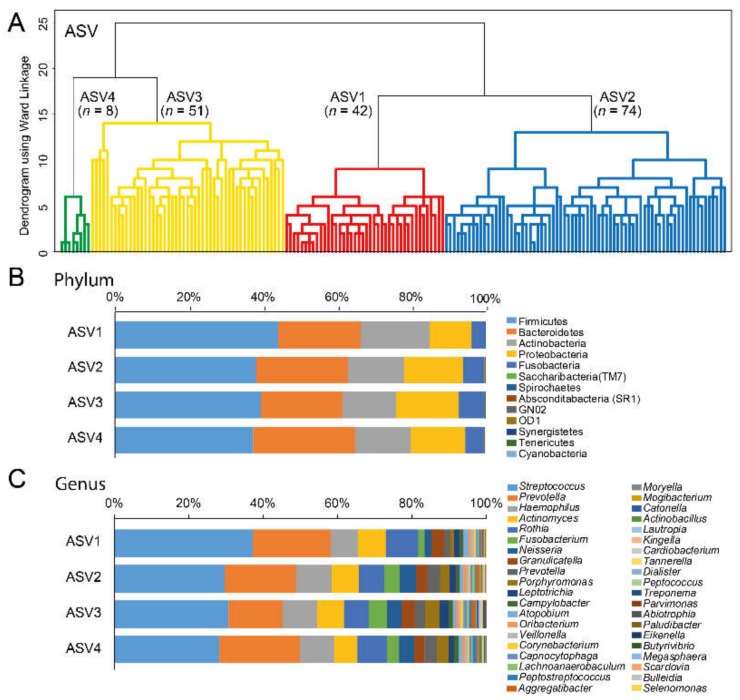
(**A**) Dendrogram from the unsupervised hierarchical cluster analysis with Ward’s method. The clusters in red and blue refer to the groups with the highest and lowest sucrose intake, respectively, when intake in the defined cluster groups was compared, (**B**) bar chart for 13 identified phyla, and (**C**) bar chart for the top 40 identified genera out of 127.

Four groups were classified from the hierarchical clustering of dichotomous ASVs (clusters ASV1-ASV4 (Figure 2A). The relative proportions of the 13 identified phyla and top 40 genera in the respective cluster group are presented in Figure 2B,C, respectively.

The ASV cluster groups differed significantly in reported sugar (*p* = 0.001) and sucrose (*p* = 0.008) intake and saliva flow rate (*p* = 0.031) (Table 2). Further, allelic variation in the *TAS1R1* (rs731024, *p* = 0.003), and the *GNAT3* (rs2074673, *p* = 0.007 and rs11760281, *p* = 0.010) genes differed significantly between the cluster groups (Table 2). None of the other gene variants or BMI or caries scores differed between cluster groups.

**Figure 3:**
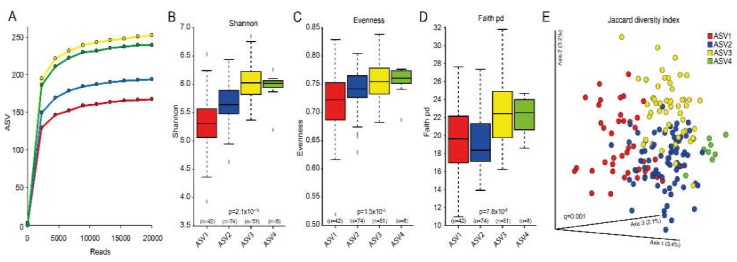
Composite figure of various aspects related to cluster groups based on dichotomous ASVs. (**A**) rarefaction curves showing number of observed ASVs by sequencing depth (reads); (**B–D**) box plots of alpha diversities by the Shannon, Evenness and Faith phylogenetic diversity (pd) indexes; (**E**) Jaccard Principal Coordinates Analysis (PCoA) plot illustrating separation of the cluster groups based on dichotomous measures. The red colour refers to the cluster with highest sucrose intake and blue refers to the lowest.

Alpha diversity was lowest for the group with the highest reported sugar intake (ASV1) when estimated as ASVs in rarefaction curves (Figure 3A), Shannon index (Figure 3B), and evenness diversities (Figure 3C), with statistically significant differences among (*p*-values from 1.5 × 10^-3^ to 1.2x10^-13^) and between groups (q-values from 1.3 × 10^-2^ to 1.7 × 10^-10^). Faith phylogenetic diversity index was less divergent between groups but still differed significantly (7.8 × 10^-5^, Figure 3D). Beta diversity differed significantly between all cluster groups (PERMANOVA *p*-value among groups and *q*-values between groups 0.001) with associated separation displayed in the Jaccard principal coordinates analysis (PCoA) plots (Figure 3E).

### 3.4. Predicted Functions in ASV Cluster Groups

Linkage of the 6171 retained 16S rRNA ASVs to pathways by KEGG functional orthology annotation predicted 10,543 KEGG orthologies. Multivariate analysis suggested a separation of the predicted function of cluster ASV1 (highest sucrose intake) from cluster ASV2 (lowest sucrose intake) and ASV3 (both *p* < 0.0006). The microbiota of cluster ASV1 (compared to both cluster ASV2 and ASV3) displayed several KEGG orthology related to starch and sucrose metabolism, and a functional enrichment of glycosidase capacity according to the STRING processing, indicating the ability to hydrolase glyosidic bonds in carbohydrates (Table S2).

### 3.5. Taxa Determination from eHOMD and Their Cluster Classification

**Figure 4:**
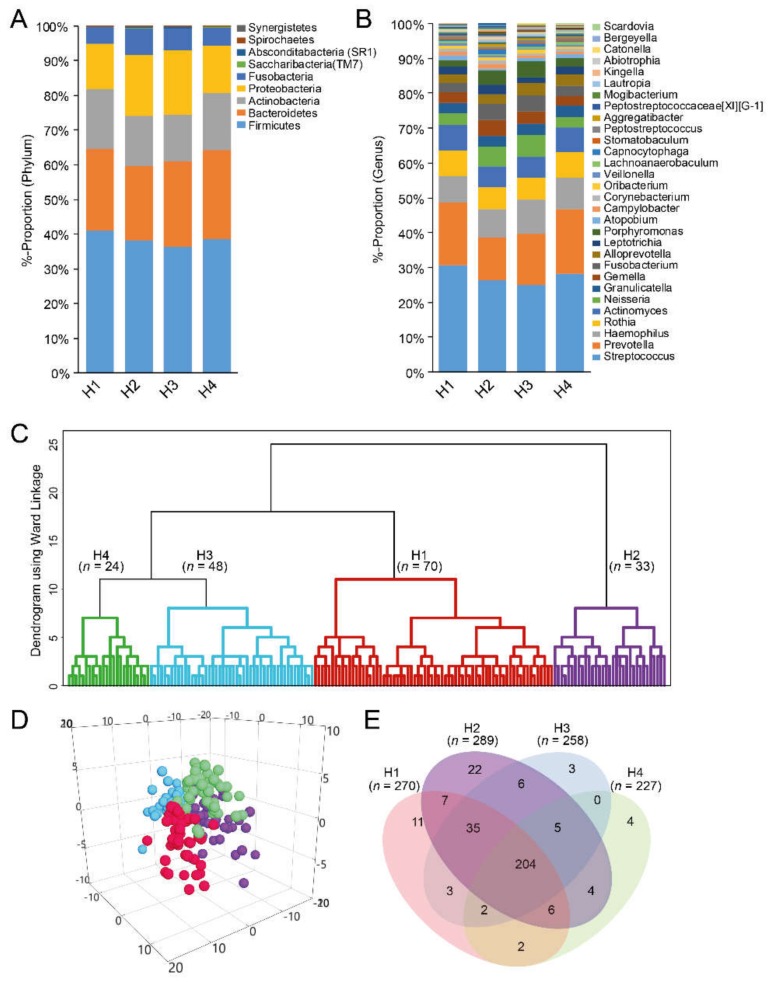
Composite figure of various aspects related to cluster groups based on eHOMD-identified taxonomic names and aggregation by species. (**A**,**B**) Bar charts showing overall relative proportions in the nine represented phyla (**A**) and top 30 genera; (**B**), as classified by the eHOMD database; (**C**) dendrogram from hierarchical cluster analysis with Ward´s method. The red section refers to the cluster with the highest sucrose intake and blue to the lowest; (**D**) PLS 3D scatter plot illustrating separation of the four cluster groups; (**E**) Venn diagrams showing the number of species detected in all four cluster groups.

The 6171 representative sequence variants matched 372 named species or unnamed phylotype gene sequences in eHOMD by the set criteria. These were in nine phyla (Figure 4A) and 85 genera. The most prevalent genera were *Streptococcus* (28.1%), and *Prevotella* (16.1%)*, Haemophilus* (8.6%)*, Rothia* (6.9%)*,* and *Actinomyces* (6.5%) (Figure 4B). The dominant species included a *Streptococcus mitis/Streptococcus infantis/Streptococcus oralis complex* (12.9%), followed by *Prevotella melaninogenica, Haemophilus parainfluenzae, Streptococcus salivarius,* and *Rothia mucilaginosa,* all of which represented >5% of total reads Table S3.

The 175 participants were classified into four groups by hierarchical clustering based on dichotomous taxa presence (clusters H1–H4, Figure 4C) with group separation, confirmed in multidimensional-PLS scatter plots (Figure 4D). Approximately half of the 372 sequences were shared among the four cluster groups (Figure 4E). Sugar (*p* = 0.006) and sucrose (*p* < 0.001) intake, and allelic variation in the *GNAT3* (rs2074673 and rs11760281) gene, differed among the groups based on dichotomous measures (Table 2).

### 3.6. Factors Associated with Belonging to the Species Level (eHOMD) Cluster Groups

PLS identified eHOMD species, host-related traits and lifestyle factors, and mutans streptococci and lactobacilli by influential culture, and classified them into the four cluster groups. Data for H1, H2 and H4 compared to H3 are shown in Table 3 and H3 from the PLS comparison including all four cluster groups simultaneously in Table S4.

Sugar and sucrose intakes were influential for being in clusters H1, H2 and H4 with comparably higher sucrose intake. Intake of milk was influential for clusters H1 and H4 (Table 2). A wide panel of species, including species in *Actinomyces* and *Selenomonas*, *Bifidobacterium dentium* and *Leptotrichia sp*. HMT498, were influential for being in cluster H2, whereas being in cluster H1 (with slightly higher mean sucrose intake) was determined by significantly fewer species, which included two species in *Actinomyces*, *Bifidobacterium longum, Scardovia wiggsiae*, *Streptococcus mutans*, two species in *Veillonella* and lactobacilli and mutans streptococci by culture. Cluster H4 also had fewer influential species than cluster H2 and included the cariogenic *Streptococcus sobrinus.*

High scores in the Healthy Diet index and for protein-related food intake were associated with membership in Cluster H3, which was the cluster with the lowest reported sucrose intake (Table S4).

### 3.7. Predicted Functions in Species Level (eHOMD) Cluster Groups

**Figure 5:**
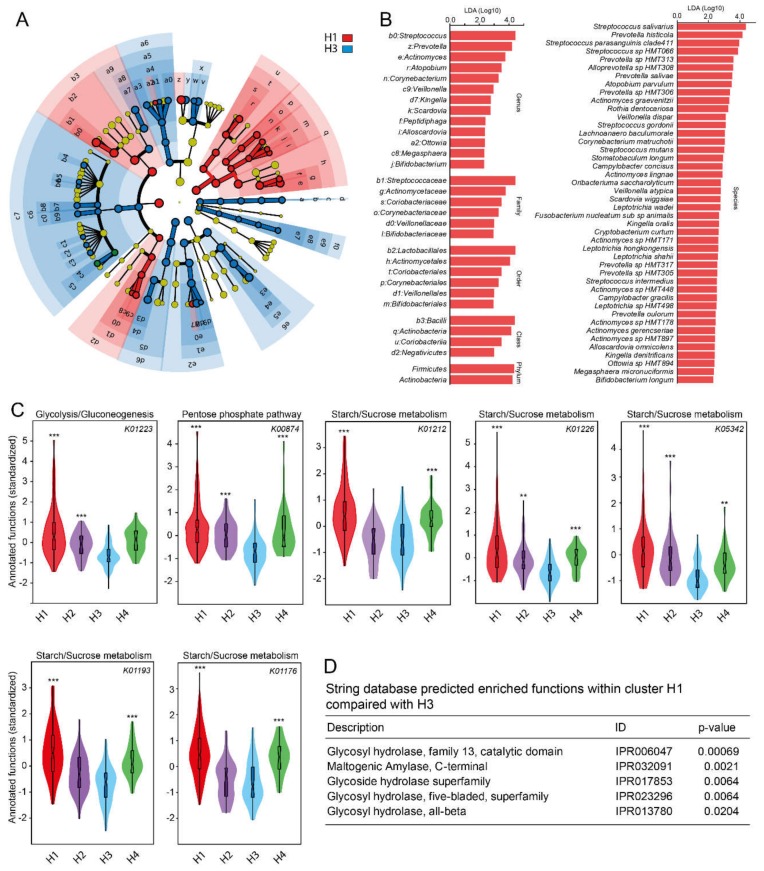
LEfSe results for Cluster H1 versus H3 with highest and lowest sucrose intake, respectively. (**A**) Cladogram showing taxonomic representation of statistically consistent differences between the two cluster groups. The analysis was based on the abundance of the 372 species and their respective family, order, class and phylum. **(B**) Histogram of the linear discriminant analysis (LDA) scores in cluster groups H1 and H3. (**C**) Violin plots with box plots for carbohydrate metabolic pathways characteristic of all four H cluster groups, and (**D**) STRING database predicted enriched functions within cluster H1 (high sucrose intake) compared with cluster H3 (lowest sucrose intake). Group comparisons were done using non-parametric (Kruskal–Wallis or Mann–Whitney U test, Bonferroni corrected p values, permutation 9999). Violin labels: ***, ** and * indicate <0.001, <0.01, and <0.05, respectively.

Predicted functions in the microbiota associated with the four HOMD-based cluster groups separated them (*p* < 0.0001), with cluster H3 (lowest sucrose intake) separated from the two groups with the highest sucrose intakes, i.e., H1 (*p* = 0.0012) and H2 (*p* = 0.027). Similar to the sequence variant-based analyses, cluster H1 displayed several KEGG functional orthology related to starch and sucrose metabolism (Figure 5C, Table S5) and a functional enrichment of the capacity to hydrolase glyosidic bonds (Figure 5D).

## 4. Discussion

This study identified groups of people defined by similar oral microbiota profiles, targeted as dichotomized amplicon species variants (ASVs) or named species and unnamed phylotypes, and then explored lifestyle and host factors which were associated with these groups. The most striking difference between groups classified by unsupervised cluster analysis was for reported sucrose intake but associations were also found for total sugar intake, saliva flow rate and allelic variations in two taste-perception-associated genes. The group who reported the highest sucrose intake had the lowest species richness, but the microbiota in clusters with a higher sucrose intake were either defined by a pamphlet of species with no clear association with carbohydrate metabolic pathways or by a microbiota enriched for acidogenic and acid-tolerant species and carbohydrate degradation metabolic pathways. The latter species included several species that were suggested to be associated with the caries disease.

The prevailing ecological plaque hypothesis [44,45] describes an ecological shift (collapse) towards the enrichment of acidogenic and acid-tolerant species in low pH environments, such as after sugar consumption [13,16,46]. The present findings are consistent with this hypothesis, as several such species were found to be enriched in two of the three cluster groups with the highest sucrose intake. Among these were species in *Actinomyces, Bifidobacterium* and *Veillonella,* and *S. wiggsiae, S. mutans*, and *S. sobrinus* with documented relevance for dental caries in small children or adults [14,44,47]. In fact, although not statistically assured, these two cluster groups tended to have had the highest mean caries experience. Conversely, the study identified a third cluster group with similar sugar intake but not characterized by enrichment of the most acidogenic and acid-tolerant species, but rather a large number of taxa including several species in *Capnocytophaga, Leptotrichia, Prevotella* and *Streptococcus*. Thus, the effects of sugar on the salivary microbiota are potentially modified by host-related factors such as innate immunity peptides and buffer functions, but there are other possible explanations, for example, if the microbiota response to sugar is regulated by inter-bacteria communication or if there was differential measurement error in the reporting of sucrose intake in the different cluster groups. Overall, the present findings are well in line with our previous finding in the same population, where *S. wiggsiae, S. mutans, B. longum, Leptotrichia sp. HMT498*, and *Selenomonas spp*. in tooth biofilm samples discriminated caries-affected from caries-free adolescents [48], and support previous experimental findings in vitro [13] and in animals [49]. A direct comparison with the results from Anderson et al. 2018 [4] may not be appropriate as they used a different 16S rRNA segment and lower similarity requirement for taxa determination.

The strength of the present study is the comparably large sample and that the study group likely represents the underlying population with minimal selection bias since the attendance rate to the public dental clinics is very high, as care is free in the targeted age group and that participants who were enrolled consecutively agreed to participate. The major weakness relates to the inherent difficulties in measuring diet using questionnaires [50]. Monitoring sugar intake may be even more challenging in the dentist´s office, since patients are aware that sugar is bad for the teeth. This may be a source of systematic measurement error which may be manifested in the low self-reported sugar intake and the limited variation compared to other studies in the country [51]. However, energy-adjustment, excluding participants who reported the most implausible energy intakes and use of robust statistical methods based on ranking rather than reported consumption, was performed to reduce the impact of this error, but it cannot completely exclude a nullifying bias from the under-reporting of sucrose intake.

Factors influential for being classified in a cluster group were assessed using multivariate PLS regression, which has the advantage of accepting correlated variables. These models were strong, but it should be noted that this is partly an effect of the fact that the cluster groups were formed by cluster analysis of bacterial taxa. However, since clustering was unsupervised, the association with sucrose intake and which specific bacteria were present in each cluster is not a consequence of clustering per se and does not prevent the comparison of phenotypic characteristics of people in the cluster groups or species that were influential for being in the groups.

An alternative model to the idea that sugar intake and, indirectly, gene variations, drives oral microbiota transformation would be that the oral microbiota per se influences the taste phenotype of the host and, accordingly, the individual’s food selection, as previously suggested [20,21,22,23]. In the present study, sucrose intake was the strongest explanatory diet factor for microbiota clustering, and supported by several in vitro and a few in vivo studies [4,7,52]. We suggest that the variant oral microbiota ecologies (here, the clusters) are a consequence of low pH due to sugar intake [42,45,52]. Thus, we suggest that the clinical implication of the present findings is that even a moderate difference in sugar intake may affect the oral microbiota into a more cariogenic composition, which may be reverted by sugar restriction. However, we cannot fully exclude that the alternative hypothesis is of relevance too.

We, and others, have reported that genetic variants located in or near genes related to taste perception and preference were associated with diet preference and caries [33], but studies have not evaluated variations in such genes in relation to the concerted oral microbiome. In this study, allelic variations in the *TAS1R1* and *GNAT3* genes were significantly associated with the pattern of oral bacteria at the group level. The *TAS1R1* gene is involved in bitter taste sensations with known associations with sweet food preferences [53], whereas the *GNAT3* gene, encoding the gustducin alpha-3 chain protein, can be involved in sweet, bitter or umami taste sensation depending on heterodimeric formations with various TAS proteins [54]. Besides taste perception, the gustducin protein functions as a sugar sensor in the gut with suggested effects on sugar absorption and metabolic syndrome [55]. Thus, hypothetically, the *GNAT3* gene may affect the oral microbiota via dietary habits or secondary effects from diet-related metabolic disorders [56].

The present study also found that under the present conditions sequencing of the v1-v3 amplicons of the 16S rDNA gene yielded significantly fewer sequences and poorer recognition of the mock species than the v3-v4 amplicons. This is in line with previous reports [57,58] and stresses the importance of taking the sequencing protocol into consideration when data are compared from different studies.

## 5. Conclusions

In conclusion, this study supported in vivo associations between sugar intake and oral microbiota ecology, but it also confirmed variations in the apparent microbiota response to sugar, highlighting the importance of considering microbes beyond the commonly studied acidogenic and acid-tolerant species and modifying host factors. The result, need to be confirmed, preferentially under controlled forms, such as in a randomized clinical trial design and with the monitoring of host pheno- and genotypical characteristics and a potential biomarker for diet, such as untargeted metabolomics.

## Figures and Tables

**Table 1:** Characteristics of the 175 participants in the study group.

	Mean (95% CI limits) or %
Women, %	51.4
Age, years	18.1 (18.0, 18.3)
BMI, kg/m^2^	22.7 (22.2, 23.2)
Overweight/obese (BMI >25), %	20.6
Smoking, %	
Present	4.4
Past	3.4
Swedish snus, %	
Present	2.9
Past	8.6
Diet^a^	
total energy, kcal/day	1 855 (1 754, 1 956)
carbohydrates, E%	40.3 (39.3, 41.4)
sugar, E%	15.0 (14.4, 15.7)
sucrose, E%	5.9 (5.6, 6.3)
protein, E%	14.1 (13.7, 14.6)
fat, E%	44.6 (43.4, 45.7)
sweet sugar snacks, daily frequency	1.1 (1.0, 1.2)
sweet non-sugar products, daily frequency	0.12 (0.08, 0.15)
milk, grams/day	209 (173, 245)
healthy diet score	12.1 (11.5, 12.7)
probiotic product latest month, %	8.3
Oral parameters^b^	
saliva flow rate, ml/min^c^	1.5 (1.4, 1.6)
proportion caries affected (DeFS>0), %	69.7
bleeding gums, %	31.1
tooth brushing ≥once a day, %	78.1
flossing or other proximal cleaning, %	25.9
any type of extra fluoride,%	6.0
mutans streptococci, median (5, 95 percentiles) for colony-forming units (CFU)/mL saliva	398 (0, 2^6^)
lactobacilli, median (5, 95 percentiles) for CFU/mL saliva	50 (0, 5^3^)
SNP variants	
*TAS1R1* rs731024 (AA), %	12.0
*GNAT3* rs2074673 (GG+GA), %	62.3
*GNAT3* rs11760281 (AA+AG), %	50.3

(**a**) 11 participants were excluded based on unrealistic reported energy intake in analyses including diet variables. Means and 95% CI are adjusted for sex, age and BMI. (**b**) For bleeding gums, 13.1%, daily brushing, 2.9%, and extra fluoride, 4.0% had missing answers. (**c**) Means and 95% CI are adjusted for sex and age.SNP, Single Nucleotide Polymorphism.

**Table 2:** Pheno- and genotypical characteristics for hierarchical cluster groups based on amplicon sequence variants (ASVs), and taxa aggregates based on identification in the eHOMD database. Underlying dendrograms are shown in Figure 2 and Figure 4, respectively. For eHOMD, only variables that differed significantly between the clusters are shown.

	Cluster by dichotomized sequence variants with ≥2 reads per ASV				
	Cluster ASV1	Cluster ASV2	Cluster ASV3	Cluster ASV4	*p*-value^a^
	*n* = 42	*n* = 74	*n* = 51	*n* = 8	
Women, %	45.2	51.4	58.8	37.5	0.497
Age	18.0 (17.7, 18.3)	18.1 (17.8, 18.3)	18.2 (17.9, 18.5)	18.8 (18.0, 19.5)	0.281
BMI^b^	23.0 (22.0, 24.0)	22.5 (21.7, 23.3)	22.6 (21.7, 23.6)	23.6 (21.3, 25.9)	0.730
Smoking, %					0.244
Present	4.8	8.2	0.0	0.0	
Past	7.1	1.4	3.9	0.0	
Swedish snus, %					0.576
Present	2.4	1.4	3.9	12.5	
Past	11.9	8.2	5.9	12.5	
Diet^c^					
total energy, kcal/day	1914 (1702, 2125)	1875 (1721, 2030)	1793 (1600, 1985)	1754 (1291, 2218)	0.591
carbohydrates, E%	42.4 (40.4, 44.6)	38.9 (37.3, 40.5)	40.5 (38.6, 42.5)	42.4 (37.5, 46.9)	0.136
sugar, E%	16.6 (15.3, 17.9)	13.6 (12.7, 14.6)	15.7 (14.6, 16.9)	16.4 (13.6, 19.2)	**0.001**
sucrose, E%	6.8 (6.1, 7.4)	5.3 (4.8, 5.7)	6.2 (5.6, 6.9)	6.2 (4.7, 7.7)	**0.008**
protein, E%	13.4 (12.5, 14.4)	14.6 (13.9, 15.3)	14.2 (13.4, 15.1)	12.3 (10.2, 14.3)	0.173
fat, E%	43.3 (40.9, 45.8)	45.3 (43.5, 47.1)	44.4 (42.2, 46.6)	44.5 (39.2, 49.8)	0.865
sweet sugar snacks, daily frequency	1.3 (1.1, 1.5)	1.0 (0.8, 1.1)	1.1 (0.9, 1.3)	1.2 (0.7, 1.7)	0.155
sweet non-sugar products, daily frequency	0.12 (0.04, 0.19)	0.12 (0.06, 0.17)	0.11 (0.04, 0.18)	0.11 (0.0, 0.27)	0.999
milk, gram/day	210 (126, 293)	208 (161, 256)	82 (0, 224)	258 (181, 334)	0.152
healthy diet score	11.8 (10.6, 13.0)	12.0 (11.1, 12.9)	12.1 (11.0, 13.1)	11.8 (11.1, 16.5)	0.629
Gene polymorphism					
*TAS1R1 (rs731024), % AA*	23.8	6.8	5.9	37.5	**0.003**
*GNAT3* (rs2074673) *% GG+GA*	54.8	65.8	72.5	12.5	**0.007**
*GNAT3* (rs11760281) *% AA+AG*	38.1	52.1	64.7	12.5	**0.010**
Oral parameters^d^					
saliva flow rate, ml/min^e^	1.3 (1.0. 1.5)	1.6 (1.5, 1.8)	1.5 (1.3, 1.7)	1.0 (0.5, 1.5)	**0.031**
DeFS^e^	6.3 (4.1, 3.2)	4.2 (2.6, 5.8)	4.4 (2.4, 6.3)	3.8 (1.0, 8.7)	0.429
bleeding gums, %	24.3	29.0	40.9	25.0	0.387
daily tooth brushing, %	68.3	87.5	79.6	75.0	0.103
extra fluoride, %	7.7	7.1	2.0	12.5	0.497
	**Cluster by dichotomous eHOMD-aggregated species with >25 reads**				
	**Cluster H1**	**Cluster H2**	**Cluster H3**	**Cluster H4**	***p*-value^a^**
	*n* = 70	*n* = 33	*n* = 48	*n* = 24	
Women, %	50.0	63.6	41.7	58.3	0.229
Sugar^c^, E%	15.8 (14.8, 16.8)	15.9 (14.3, 17.4)	13.2 (12.1, 14.4)	15.6 (13.9, 17.2)	**0.006**
Sucrose^c^, E%	6.5 (6.0, 7.0)	6.4 (5.7, 7.2)	4.9 (4.3, 5.5)	6.0 (5.2, 6.9)	**<0.001**
SNP variants					
*GNAT3* (rs2074673) *% GG+GA*	56.5	84.8	66.7	41.7	**0.005**
*GNAT3* (rs11760281) *% AA+AG*	47.8	72.2	54.2	20.8	**0.001**

All tests were non-parametric. Adjustment for sex and age did not affect the results. (**a**) 11 participants were excluded in analyses including diet variables due to unrealistic reported energy intake. Means and 95% CI are adjusted for sex, age and BMI. (**b**) For bleeding gums, 13.1%, daily brushing,2.9%, and extra fluoride, 4.0% had missing answers, respectively. (**c**) Means and 95% CI are adjusted for sex and age. *p*-values in bold are considered significant. ASV, amplicon sequence variant.

**Table 3:** Species significantly influential for being classified into cluster H1, H2 or H4, respectively, when compared with cluster H3. Species in bold are known to be acidogenic and acid-tolerant species. Data for cluster H3 (lowest sucrose intake) against the three other clusters simultaneously are found in Table S4.

Cluster H1 (*n* = 70)	Cluster H2 (*n* = 33)	Cluster H4 (*n* = 24)
model R^2^ = 50%, Q^2^ = 50%	model R^2^ = 84%, Q^2^ = 63%	model R^2^ = 80, Q^2^ = 53
sucrose intake = 6.5 E%	sucrose intake 6.4 E%	sucrose intake 6.0 E%
DeFS = 5.8	DeFS = 4.5	DeFS = 5.1
sucrose, E%	sucrose, E%	sucrose, E%
sugar, E%	sugar, E%	sugar, Eproc
milk 3%		milk, 1,5%
monosaccharides, E%		
***Actinomyces sp. HMT171***	***Actinomyces israelii***	***Actinomyces odontolyticus***
***Actinomyces sp. HMT178***	***Actinomyces massiliensis***	***Actinomyces sp. HMT171***
*Alloprevotella sp. HMT308*	***Actinomyces sp. HMT171***	***Actinomyces sp. HMT448***
*Alloscardovia omnicolens*	***Actinomyces sp. HMT178***	*Aggregatibacter sp. HMT458*
***Bifidobacterium longum***	***Actinomyces sp. HMT897***	*Alloprevotella sp. HMT308*
*Capnocytophaga sp. HMT326*	*Aggregatibacter sp. HMT458*	*Bacteroidales [G-2] bacterium HMT274*
*Capnocytophaga sp. HMT902*	*Aggregatibacter sp. HMT949*	*Bacteroidetes [G-5] bacterium HMT505*
*Dietzia cinnamea*	*Alloprevotella sp. HMT912*	*Bacteroidetes [G-5] bacterium HMT511*
*Lachnoanaerobaculum orale*	*Alloprevotella sp. HMT913*	*Capnocytophaga sp. HMT326*
***lactobacilli, culture***	*Atopobium rimae*	*Capnocytophaga sp. HMT338*
*Leptotrichia wadei*	*Bacteroidales [G-2] bacterium HMT274*	*Corynebacterium singulare*
*Megasphaera micronuciformis*	*Bacteroidetes [G-3] bacterium HMT281*	*Dialister invisus*
***mutans streptococci, culture***	*Bacteroidetes [G-5] bacterium HMT511*	*Dialister pneumosintes*
*Olsenella sp. HMT807*	*Bergeyella sp. HMT206*	*Fusobacterium nucleatum ssp. animalis*
*Peptostreptococcaceae [XI][G-7] yurii*	*Bergeyella sp. HMT907*	*Granulicatella elegans*
*Prevotella histicola*	***Bifidobacterium dentium***	*Haemophilus parahaemolyticus*
*Prevotella sp. HMT305*	*Butyrivibrio sp. HMT080*	*Kingella oralis*
*Prevotella sp. HMT306*	*Campylobacter gracilis*	*Lachnoanaerobaculum orale*
*Prevotella sp. HMT313*	*Capnocytophaga granulosa*	*Lactobacillus crispatus*
*Prevotella sp. HMT317*	*Capnocytophaga haemolytica*	*Leptotrichia sp. HMT221*
*Scardovia wiggsiae*	*Capnocytophaga ochracea*	*Leptotrichia wadei*
*Stomatobaculum longum*	*Capnocytophaga sp. HMT326*	*Megasphaera micronuciformis*
*Streptococcus intermedius*	*Capnocytophaga sp. HMT332*	*Mycoplasma faucium*
*Streptococcus mutans*	*Capnocytophaga sp. HMT338*	*Neisseria bacilliformis*
*Streptococcus parasanguinis clade411*	*Capnocytophaga sp. HMT903*	*Olsenella sp. HMT807*
***Veillonella atypica***	*Cardiobacterium valvarum*	*Peptococcus sp. HMT167*
***Veillonella dispar***	*Catonella sp. HMT164*	*Peptostreptococcaceae [XI][G-5] saphenum*
	*Dialister pneumosintes*	*Peptostreptococcaceae [XI][G-9] brachy*
	*Eikenella corrodens*	*Porphyromonas endodontalis*
	*Fusobacterium hwasookii*	*Prevotella denticola*
	*Fusobacterium naviforme*	*Prevotella histicola*
	*Fusobacterium nucleatum subsp. animalis*	*Prevotella intermedia*
	*Fusobacterium nucleatum subsp. polymorphum*	*Prevotella sp. HMT305*
	*Fusobacterium sp. HMT204*	*Prevotella sp. HMT306*
	*Gemella morbillorum*	*Prevotella sp. HMT317*
	*Haemophilus haemolyticus*	*Saccharibacteria (TM7) [G-5] bacterium HMT356*
	*Johnsonella sp. HMT166*	***Scardovia wiggsiae***
	*Kingella denitrificans*	***Streptococcus mutans***
	*Kingella oralis*	*Streptococcus parasanguinis clade 411*
	*Kingella sp. HMT012*	***Streptococcus sobrinus***
	*Lachnoanaerobaculum saburreum*	*Streptococcus sp. HMT057*
	*Leptotrichia buccalis*	*Tannerella forsythia*
	*Leptotrichia shahii*	*Treponema denticola*
	*Leptotrichia sp. HMT219*	*Treponema lecithinolyticum*
	*Leptotrichia sp. HMT223*	*Treponema socranskii*
	*Leptotrichia sp. HMT392*	*Treponema sp. HMT237*
	*Leptotrichia sp. HMT498*	***Veillonella atypica***
	*Leptotrichia wadei*	***Veillonella dispar***
	*Mycoplasma salivarium*	*GNAT3* (rs11760281
	*Olsenella sp. HMT807*	
	*Oribacterium sp. HMT078*	
	*Ottowia sp. HMT894*	
	*Parvimonas micra*	
	*Peptococcus sp. HMT167*	
	*Peptostreptococcaceae [XI][G-5] saphenum*	
	*Peptostreptococcaceae [XI][G-7] bacterium HMT081*	
	*Peptostreptococcaceae [XI][G-7] yurii*	
	*Porphyromonas catoniae*	
	*Porphyromonas sp. HMT275*	
	*Porphyromonas sp. HMT278*	
	*Prevotella fusca*	
	*Prevotella intermedia*	
	*Prevotella maculosa*	
	*Prevotella micans*	
	*Prevotella nigrescens*	
	*Prevotella oulorum*	
	*Prevotella pleuritidis*	
	*Prevotella saccharolytica*	
	*Prevotella sp. HMT300*	
	*Prevotella sp. HMT301*	
	*Prevotella sp. HMT317*	
	*Prevotella sp. HMT472*	
	*Prevotella sp. HMT475*	
	*Rothia aeria*	
	*Saccharibacteria (TM7) [G-1]bacterium HMT348*	
	*Saccharibacteria (TM7) [G-5] bacterium HMT356*	
	*Selenomonas noxia*	
	*Stomatobaculum longum*	
	*Streptococcus constellatus*	
	*Streptococcus gordonii*	
	*Streptococcus intermedius*	
	*Streptococcus parasanguinis clade 411*	
	*Tannerella forsythia*	
	*Treponema socranskii*	
	*Treponema sp. HMT237*	
	*Treponema sp. HMT246*	
	*Treponema sp. HMT262*	

Taxa in Cluster H1 (high sucrose intake) compared with H3 (lowest sugar intake) from LEfSe analysis is further illustrated in a clade diagram (Figure 5A) with effect sizes in an LDA histogram (Figure 5B). Species in the phyla *Streptococcus* and *Actinomyces* were associated with H1. At the species level, largely the same species as identified by PLS appeared. The strongest effect sizes (LDA scores of about 4) were seen for species in the genera *Streptococcus* and *Prevotella* (Figure 5B).

## Supplementary Materials

The following are available online at https://www.mdpi.com/2072-6643/12/3/681/s1. Table S1: Taxa detected by Illumina MISeq sequencing of the v1–v3 versus the v3–v4 variable regions of 16S rDNA. Identities are from eHOMD blasting at 98.5% identity and cut-off at 25 reads per species. Table S2: Partial least square regression analysis (PLS-DA) was used to identify bacterial functions differentiating between ASV cluster 2, with the lowest sucrose intake (sugar 13.6 E%, sucrose 5.3E%), to that of cluster ASV 1, 3, and 4 with higher sucrose intakes. Bacterial functions were predicted by Phylogenetic Investigation of Communities by Reconstruction of Unobserved States (PICRUSt 2) in QIIME 2 and Kyoto Encyclopedia of Genes and Genomes (KEGG orthology, KO). Bold labelled functions are linked to Carbohydrate metabolism (e.g., starch, sucrose, and glycolysis). Table S3: Prevalence of 372 species identified from the eHOMD database at 98.5% identity and represented by at least 25 reads. Data are for cluster groups from hierarchical clustering of dichotomous variables. *p*-values for the prevalences are from Chi-square analyses. Species and p-values in bold are statistically significant at FDR 0.05. Table S4: Bacteria and other host-associated variables with a statistically significant association (PLS correlation coefficients with 95% CI that does not include 0) in PLS modelling. Cluster H3 (the cluster with the lowest reported sucrose intake) was modelled against cluster H1, H2, and H4 individually or together. Sucrose intake (E%) and caries (DeFS) are group mean values standardized for sex, age and BMI and sex and age, respectively. Variables are ordered by taxa names and other host factors at the end. Species in bold are species that commonly are referred to as acidophilic species. Table S5: Multivariate analysis (PLS-DA) was used to identify bacterial functions differentiating between cluster H3, with the lowest sucrose intake) to that of clusters H1, H2, and H4 with higher sucrose intake. Phylogenetic Investigation of Communities by Reconstruction of Unobserved States (PICRUSt 2) in QIIME 2 and Kyoto Encyclopedia of Genes and Genomes (KEGG orthology, KO) predicted bacterial functions. Functions in bold are linked to carbohydrate metabolism (e.g., starch, sucrose, and glycolysis).
