# Supplementary material for: Oral Microbiota Profile Associates with Sugar Intake and Taste Preference Genes
Source: Nutrients. 2020 Mar 3;12(3):681. doi: 10.3390/nu12030681 (PMC7146170; doi:10.3390/nu12030681)
Supplement: Supplementary file 1 [file nutrients-12-00681-s001.zip › Table S1.docx]

**Table S1.** Taxa detected by Illumina MISeq sequencing of the v1-v3 versus the v3-v4 variable regions of 16S rDNA. Identities are from eHOMD blasting at 98.5% identity and cut-off at 25 reads per species.

| Taxa detection | | |
| --- | --- | --- |
| among both v1-v3 and v3-v4 sequences | among v1-v3 sequences only | among V3-V4 sequences only |
| *Absconditabacteria (SR1) [G-1]* HMT345 | *Campylobacter showae* | *Abiotrophia defectiva* |
| *Absconditabacteria (SR1) [G-1]* HMT874 | *Capnocytophaga sp.* HMT878 | *Actinomyces dentalis* |
| *Absconditabacteria (SR1) [G-1]* HMT875 | *Freti sp. HMT359* | *Actinomyces gerencseriae* |
| *Acidipropioni acidifaciens* | *Freti sp. HMT362* | *Actinomyces israelii* |
| *Actinomyces georgiae* | *Fuso sp. HMT204* | *Actinomyces johnsonii* |
| *Actinomyces graevenitzii* | *Mogi diversum* | *Actinomyces lingnae* |
| *Actinomyces odontolyticus* | *Propionibacteriaceae [G-1]* HMT915 | *Actinomyces massiliensis* |
| *Actinomyces sp.* HMT169 | *Pseudopropioni sp.* HMT194 | *Actinomyces naeslundii* |
| *Actinomyces sp.* HMT172 | *Saccharibacteria (TM7) [G-8]* HMT955 | *Actinomyces oris* |
| *Actinomyces sp.* HMT175 |  | *Actinomyces sp.* HMT170 |
| *Actinomyces sp.* HMT180 |  | *Actinomyces sp.* HMT171 |
| *Aggregatibacter sp.* HMT458 |  | *Actinomyces sp.* HMT178 |
| *Aggregatibacter sp.* HMT898 |  | *Actinomyces sp.* HMT414 |
| *Alloprevotella sp.* HMT308 |  | *Actinomyces sp.* HMT448 |
| *Alloprevotella sp.* HMT914 |  | *Actinomyces sp.* HMT525 |
| *Alloscardovia omnicolens* |  | *Actinomyces sp.* HMT877 |
| *Atopobium parvulum* |  | *Actinomyces sp.* HMT896 |
| *Atopobium rimae* |  | *Actinomyces sp.* HMT897 |
| *Bergeyella sp.* HMT206 |  | *Actinomyces timonensis* |
| *Campylobacter concisus* |  | *Aggregatibacter actinomycetemcomitans* |
| *Campylobacter gracilis* |  | *Aggregatibacter aphrophilus* |
| *Campylobacter rectus* |  | *Aggregatibacter paraphrophilus* |
| *Campylobacter sp.* HMT044 |  | *Aggregatibacter segnis* |
| *Capnocytophaga gingivalis* |  | *Aggregatibacter sp.* HMT513 |
| *Capnocytophaga granulosa* |  | *Aggregatibacter sp.* HMT949 |
| *Capnocytophaga leadbetteri* |  | *Alloprevotella rava* |
| *Capnocytophaga sp.* HMT326 |  | *Alloprevotella sp.* HMT473 |
| *Capnocytophaga sp.* HMT332 |  | *Alloprevotella sp.* HMT912 |
| *Capnocytophaga sp.* HMT338 |  | *Alloprevotella sp.* HMT913 |
| *Capnocytophaga sp.* HMT901 |  | *Alloprevotella tannerae* |
| *Capnocytophaga sputigena* |  | *Atopobium sp.* HMT199 |
| *Catonella morbi* |  | *Bacteroidaceae [G-1]* HMT272 |
| *Clostridiales [F-1][G-1]* HMT093 |  | *Bacteroidales [G-2]* HMT274 |
| *Coryne matruchotii* |  | *Bacteroidetes [G-3]* HMT280 |
| *Crypto curtum* |  | *Bacteroidetes [G-3]* HMT281 |
| *Dietzia cinnamea* |  | *Bacteroidetes [G-3]* HMT365 |
| *Eikenella corrodens* |  | *Bacteroidetes [G-3] HMT503* |
| *Freti fastidiosum* |  | *Bacteroidetes [G-5] HMT505* |
| *Freti sp.* HMT360 |  | *Bacteroidetes [G-5] HMT507* |
| *Fuso gonidiaformans* |  | *Bacteroidetes [G-5] HMT511* |
| *Fuso hwasookii* |  | *Bacteroidetes [G-7] HMT911* |
| *Fuso naviforme* |  | *Bergeyella sp.* HMT322 |
| *Fuso necrophorum* |  | *Bergeyella sp.* HMT900 |
| *Fuso nucleatum subsp. animalis* |  | *Bergeyella sp.* HMT907 |
| *Fuso nucleatum subsp. nucleatum* |  | *Bergeyella sp.* HMT931 |
| *Fuso nucleatum subsp. polymorphum* |  | *Bifido breve* |
| *Fuso nucleatum subsp. vincentii* |  | *Bifido dentium* |
| *Fuso periodonticum* |  | *Bifido longum* |
| *Fuso sp.* HMT203 |  | *Butyrivibrio sp.* HMT080 |
| *Fuso sp.* HMT248 |  | *Butyrivibrio sp.* HMT455 |
| *Fuso sp.* HMT370 |  | *Campylobacter curvus* |
| *Gemella haemolysans* |  | *Capnocytophaga haemolytica* |
| *Gemella morbillorum* |  | *Capnocytophaga ochracea* |
| *Gemella sanguinis* |  | *Capnocytophaga sp.* HMT323 |
| *Gracilibacteria (GN02) [G-1]* HMT871 |  | *Capnocytophaga sp.* HMT324 |
| *Gracilibacteria (GN02) [G-1]* HMT872 |  | *Capnocytophaga sp.* HMT335 |
| *Granulicatella adiacens* |  | *Capnocytophaga sp.* HMT336 |
| *Granulicatella elegans* |  | *Capnocytophaga sp.* HMT380 |
| *Haemophilus haemolyticus* |  | *Capnocytophaga sp.* HMT412 |
| *Haemophilus parahaemolyticus* |  | *Capnocytophaga sp.* HMT863 |
| *Haemophilus parainfluenzae* |  | *Capnocytophaga sp.* HMT864 |
| *Haemophilus pittmaniae* |  | *Capnocytophaga sp.* HMT902 |
| *Haemophilus sp.* HMT036 |  | *Capnocytophaga sp.* HMT903 |
| *Haemophilus sp.* HMT908 |  | *Cardio hominis* |
| *Haemophilus sputorum* |  | *Cardio valvarum* |
| *Kingella denitrificans* |  | *Catonella sp.* HMT451 |
| *Kingella oralis* |  | *Coryne durum* |
| *Kocuria rhizophila* |  | *Coryne singulare* |
| *Lachnoanaerobaculum orale* |  | *Desulfobulbus sp.* HMT041 |
| *Lachnoanaerobaculum saburreum* |  | *Dialister invisus* |
| *Lachnospiraceae [G-3]* HMT100 |  | *Dialister micraerophilus* |
| *Lautropia mirabilis* |  | *Dialister pneumosintes* |
| *Leptotrichia buccalis* |  | *Dialister sp.* HMT119 |
| *Leptotrichia goodfellowii* |  | *Filifactor alocis* |
| *Leptotrichia hofstadii* |  | *Gardnerella vaginalis* |
| *Leptotrichia hongkongensis* |  | *Gemella bergeri* |
| *Leptotrichia shahii* |  | *Gemella sp.* HMT928 |
| *Leptotrichia sp.* HMT212 |  | *Haemophilus aegyptius* |
| *Leptotrichia sp.* HMT215 |  | *Haemophilus influenzae* |
| *Leptotrichia sp.* HMT218 |  | *Haemophilus paraphrohaemolyticus* |
| *Leptotrichia sp.* HMT219 |  | *Johnsonella ignava* |
| *Leptotrichia sp.* HMT221 |  | *Johnsonella sp.* HMT166 |
| *Leptotrichia sp.* HMT223 |  | *Kingella kingae* |
| *Leptotrichia sp.* HMT225 |  | *Kingella sp.* HMT012 |
| *Leptotrichia sp.* HMT392 |  | *Kingella sp.* HMT459 |
| *Leptotrichia sp.* HMT417 |  | *Kingella sp. HMT932* |
| *Leptotrichia sp.* HMT463 |  | *Lachnoanaerobaculum umeaense* |
| *Leptotrichia sp.* HMT498 |  | *Lachnospiraceae [G-2] HMT088* |
| *Leptotrichia sp.* HMT847 |  | *Lachnospiraceae [G-2] HMT096* |
| *Leptotrichia sp.* HMT909 |  | *Lachnospiraceae [G-8] HMT500* |
| *Leptotrichia wadei* |  | *Lactobacillus casei* |
| *Mogi timidum* |  | *Lactobacillus crispatus* |
| *Neisseria elongata* |  | *Lactobacillus fermentum* |
| *Neisseria perflava* |  | *Lactobacillus gasseri* |
| *Neisseria subflava* |  | *Lactobacillus panis* |
| *Olsenella sp.* HMT807 |  | *Lactobacillus paracasei* |
| *Olsenella uli* |  | *Lactobacillus plantarum* |
| *Ori asaccharolyticum* |  | *Lactobacillus reuteri clade 938* |
| *Ori parvum* |  | *Lactobacillus salivarius* |
| *Ori sinus* |  | *Lactobacillus vaginalis* |
| *Peptostreptococcaceae [XI][G-1] sulci* |  | *Lactococcus lactis* |
| *Peptostreptococcaceae [XI][G-9] brachy* |  | *Leptotrichia sp.* HMT879 |
| *Peptostreptococcus stomatis* |  | *Megasphaera micronuciformis* |
| *Porphyromonas catoniae* |  | *Mogi vescum* |
| *Porphyromonas gingivalis* |  | *Moraxella sp.* HMT276 |
| *Porphyromonas pasteri* |  | *Mycoplasma faucium* |
| *Porphyromonas sp.* HMT275 |  | *Mycoplasma hominis* |
| *Prevotella denticola* |  | *Mycoplasma orale* |
| *Prevotella histicola* |  | *Mycoplasma salivarium* |
| *Prevotella maculosa* |  | *Neisseria bacilliformis* |
| *Prevotella melaninogenica* |  | *Neisseria cinerea* |
| *Prevotella nanceiensis* |  | *Neisseria flavescens* |
| *Prevotella oulorum* |  | *Neisseria macacae* |
| *Prevotella pallens* |  | *Neisseria oralis* |
| *Prevotella salivae* |  | *Neisseria sp.* HMT018 |
| *Prevotella sp.* HMT306 |  | *Neisseria sp.* HMT499 |
| *Prevotella sp.* HMT313 |  | *Ori sp.* HMT078 |
| *Prevotella sp.* HMT314 |  | *Ottowia sp.* HMT894 |
| *Prevotella sp.* HMT317 |  | *Parascardovia denticolens* |
| *Pseudopropioni propionicum* |  | *Parvimonas micra* |
| *Rothia aeria* |  | *Parvimonas sp.* HMT110 |
| *Rothia dentocariosa* |  | *Parvimonas sp.* HMT393 |
| *Rothia mucilaginosa* |  | *Peptidiphaga gingivicola* |
| *Ruminococcaceae [G-1]* HMT075 |  | *Peptidiphaga sp.* HMT183 |
| *Ruminococcaceae [G-2] HMT085* |  | *Peptococcus sp.* HMT167 |
| *Saccharibacteria (TM7) [G-1] HMT346* |  | *Peptococcus sp.* HMT168 |
| *Saccharibacteria (TM7) [G-1] HMT347* |  | *Peptoniphilus lacrimalis* |
| *Saccharibacteria (TM7) [G-1] HMT348* |  | *Peptostreptococcaceae [XI][G-2] HMT091* |
| *Saccharibacteria (TM7) [G-1] HMT349* |  | *Peptostreptococcaceae [XI][G-4] HMT369* |
| *Saccharibacteria (TM7) [G-1] HMT352* |  | *Peptostreptococcaceae [XI][G-5] HMT493* |
| *Saccharibacteria (TM7) [G-1] HMT488* |  | *Peptostreptococcaceae [XI][G-5] saphenum* |
| *Saccharibacteria (TM7) [G-1] HMT869* |  | *Peptostreptococcaceae [XI][G-6] nodatum* |
| *Saccharibacteria (TM7) [G-1] HMT952* |  | *Peptostreptococcaceae [XI][G-7] HMT081* |
| *Saccharibacteria (TM7) [G-2] HMT350* |  | *Peptostreptococcaceae [XI][G-7] HMT922* |
| *Saccharibacteria (TM7) [G-3] HMT351* |  | *Peptostreptococcaceae [XI][G-7] yuri* |
| *Saccharibacteria (TM7) [G-5] HMT356* |  | *Porphyromonas asaccharolytica* |
| *Slackia exigua* |  | *Porphyromonas endodontalis* |
| *Sneathia amni* |  | *Porphyromonas sp.* HMT277 |
| *Solo moorei* |  | *Porphyromonas sp.* HMT278 |
| *Stomatobaculum sp.* HMT097 |  | *Porphyromonas sp.* HMT284 |
| *Streptococcus australis* |  | *Porphyromonas sp.* HMT285 |
| *Streptococcus cristatus clade 578* |  | *Porphyromonas sp.* HMT930 |
| *Streptococcus infantis clade 638* |  | *Porphyromonas uenonis* |
| *Streptococcus parasanguinis clade 411* |  | *Prevotella aurantiaca* |
| *Streptococcus parasanguinis clade 721* |  | *Prevotella baroniae* |
| *Streptococcus salivarius* |  | *Prevotella buccae* |
| *Streptococcus sanguinis* |  | *Prevotella dentalis* |
| *Streptococcus sp. HMT074* |  | *Prevotella enoeca* |
| *Tannerella sp.* HMT286 |  | *Prevotella fusca* |
|  |  | *Prevotella intermedia* |
|  |  | *Prevotella loescheii* |
|  |  | *Prevotella marshii* |
|  |  | *Prevotella micans* |
|  |  | *Prevotella multiformis* |
|  |  | *Prevotella nigrescens* |
|  |  | *Prevotella oralis* |
|  |  | *Prevotella oris* |
|  |  | *Prevotella pleuritidis* |
|  |  | *Prevotella saccharolytica* |
|  |  | *Prevotella scopos* |
|  |  | *Prevotella shahii* |
|  |  | *Prevotella sp.* HMT292 |
|  |  | *Prevotella sp.* HMT300 |
|  |  | *Prevotella sp.* HMT301 |
|  |  | *Prevotella sp.* HMT304 |
|  |  | *Prevotella sp.* HMT305 |
|  |  | *Prevotella sp.* HMT309 |
|  |  | *Prevotella sp.* HMT315 |
|  |  | *Prevotella sp.* HMT443 |
|  |  | *Prevotella sp.* HMT472 |
|  |  | *Prevotella sp.* HMT475 |
|  |  | *Prevotella sp.* HMT526 |
|  |  | *Prevotella sp.* HMT942 |
|  |  | *Prevotella veroralis* |
|  |  | *Saccharibacteria (TM7) [G-6] HMT870* |
|  |  | *Scardovia wiggsiae* |
|  |  | *Selenomonas artemidis* |
|  |  | *Selenomonas dianae* |
|  |  | *Selenomonas noxia* |
|  |  | *Selenomonas sp.* HMT126 |
|  |  | *Selenomonas sp.* HMT136 |
|  |  | *Selenomonas sp.* HMT137 |
|  |  | *Selenomonas sp.* HMT138 |
|  |  | *Selenomonas sp.* HMT478 |
|  |  | *Selenomonas sputigena* |
|  |  | *Shuttleworthia satelles* |
|  |  | *Simonsiella muelleri* |
|  |  | *Staphylococcus aureus* |
|  |  | *Staphylococcus capitis* |
|  |  | *Stomatobaculum longum* |
|  |  | *Streptococcus anginosus* |
|  |  | *Streptococcus constellatus* |
|  |  | *Streptococcus gordonii* |
|  |  | *Streptococcus intermedius* |
|  |  | *Streptococcus lactarius* |
|  |  | *Streptococcus mutans* |
|  |  | *Streptococcus peroris* |
|  |  | *Streptococcus pneumoniae* |
|  |  | *Streptococcus sobrinus* |
|  |  | *Streptococcus thermophilus* |
|  |  | *Tannerella forsythia* |
|  |  | *Tannerella sp.* HMT808 |
|  |  | *Tannerella sp.* HMT916 |
|  |  | *Treponema denticola* |
|  |  | *Treponema lecithinolyticum* |
|  |  | *Treponema maltophilum* |
|  |  | *Treponema medium* |
|  |  | *Treponema socranskii* |
|  |  | *Treponema sp.* HMT226 |
|  |  | *Treponema sp.* HMT230 |
|  |  | *Treponema sp.* HMT231 |
|  |  | *Treponema sp.* HMT234 |
|  |  | *Treponema sp.* HMT235 |
|  |  | *Treponema sp.* HMT237 |
|  |  | *Treponema sp.* HMT238 |
|  |  | *Treponema sp.* HMT246 |
|  |  | *Treponema sp.* HMT251 |
|  |  | *Treponema sp.* HMT253 |
|  |  | *Treponema sp.* HMT256 |
|  |  | *Treponema sp.* HMT257 |
|  |  | *Treponema sp.* HMT258 |
|  |  | *Treponema sp.* HMT262 |
|  |  | *Treponema sp.* HMT951 |
|  |  | *Treponema vincentii* |
|  |  | *Veillonella atypica* |
|  |  | *Veillonella dispar* |
|  |  | *Veillonella parvula* |
|  |  | *Veillonella rogosae* |
|  |  | *Veillonella sp.* HMT780 |
|  |  | *Veillonella sp.* HMT917 |
