# Supplementary material for: Oral Microbiota Profile Associates with Sugar Intake and Taste Preference Genes
Source: Nutrients. 2020 Mar 3;12(3):681. doi: 10.3390/nu12030681 (PMC7146170; doi:10.3390/nu12030681)
Supplement: Supplementary file 1 [file nutrients-12-00681-s001.zip › Table S2.docx]

| **Table S2.** Partial least square regression analysis (PLS-DA) was used to identify bacterial functions differentiating between ASV cluster 2, with the lowest sucrose intake (sugar 13.6 E%, sucrose 5.3 E%), to that of cluster ASV1, ASV 3, and ASV 4 with higher sucrose intakes. Bacterial functions were predicted by Phylogenetic Investigation of Communities by Reconstruction of Unobserved States (PICRUSt 2) in QIIME 2 and Kyoto Encyclopedia of Genes and Genomes (KEGG orthology, KO). Bold labelled functions are linked to Carbohydrate metabolism (e.g. starch, sucrose, and glycolysis). | | | | | | | |  |
| --- | --- | --- | --- | --- | --- | --- | --- | --- |
| **Cluster ASV1 (n=42, R2=39.6%, Q2=24.0%, sugar 16.6E%, sucrose 6.8E%, DeFS 6.3)** | | | | | | | |  |
| **KO** | **VIP** | **95%CI** | **Level 1** | **Level 2** | | **Level 3** | |  |
| K00818 | 2.4 | 1.7 | Metabolism | Amino Acid Metabolism | | Amino acid related enzymes | |  |
| K00547 | 2.4 | 1.5 | Metabolism | Amino Acid Metabolism | | Cysteine and methionine metabolism | |  |
| K01243 | 2.3 | 1.0 | Metabolism | Amino Acid Metabolism | | Cysteine and methionine metabolism | |  |
| K08969 | 2.3 | 1.4 | Metabolism | Amino Acid Metabolism | | Cysteine and methionine metabolism | |  |
| K00841 | 2.2 | 1.0 | Metabolism | Amino Acid Metabolism | | Lysine biosynthesis | |  |
| K01439 | 2.1 | 1.0 | Metabolism | Amino Acid Metabolism | | Lysine biosynthesis | |  |
| K01817 | 2.1 | 0.8 | Metabolism | Amino Acid Metabolism | | Phenylalanine, tyrosine and tryptophan biosynthesis | |  |
| K04092 | 2.3 | 1.1 | Metabolism | Amino Acid Metabolism | | Phenylalanine. tyrosine and tryptophan biosynthesis | |  |
| K04518 | 2.3 | 1.1 | Metabolism | Amino Acid Metabolism | | Phenylalanine. tyrosine and tryptophan biosynthesis | |  |
| K00817 | 2.2 | 1.3 | Metabolism | Biosynthesis of Other Secondary Metabolites | | Tropane, piperidine and pyridine alkaloid biosynthesis | |  |
| **K01813** | **2.5** | **1.3** | **Metabolism** | **Carbohydrate Metabolism** | | **Fructose and mannose metabolism** | |  |
| **K01629** | **2.3** | **0.6** | **Metabolism** | **Carbohydrate Metabolism** | | **Fructose and mannose metabolism** | |  |
| **K01223** | **2.5** | **1.1** | **Metabolism** | **Carbohydrate Metabolism** | | **Glycolysis / Gluconeogenesis** | |  |
| **K00131** | **2.2** | **1.5** | **Metabolism** | **Carbohydrate Metabolism** | | **Glycolysis / Gluconeogenesis** | |  |
| **K01621** | **2.5** | **0.6** | **Metabolism** | **Carbohydrate Metabolism** | | **Pentose phosphate pathway** | |  |
| **K01839** | **2.1** | **1.0** | **Metabolism** | **Carbohydrate Metabolism** | | **Pentose phosphate pathway** | |  |
| **K01624** | **2.1** | **0.5** | **Metabolism** | **Carbohydrate Metabolism** | | **Pentose phosphate pathway** | |  |
| **K00874** | **2.1** | **1.0** | **Metabolism** | **Carbohydrate Metabolism** | | **Pentose phosphate pathway** | |  |
| **K05343** | **2.2** | **1.4** | **Metabolism** | **Carbohydrate Metabolism** | | **Starch and sucrose metabolism** | |  |
| **K01182** | **2.1** | **0.9** | **Metabolism** | **Carbohydrate Metabolism** | | **Starch and sucrose metabolism** | |  |
| **K01226** | **2.6** | **1.2** | **Metabolism** | **Carbohydrate Metabolism** | | **Starch and sucrose metabolism** | |  |
| **K00689** | **2.4** | **1.6** | **Metabolism** | **Carbohydrate Metabolism** | | **Starch and sucrose metabolism** | |  |
| **K01193** | **2.3** | **1.1** | **Metabolism** | **Carbohydrate Metabolism** | | **Starch and sucrose metabolism** | |  |
| **K01812** | **2.2** | **0.9** | **Metabolism** | **Carbohydrate Metabolism** | | **Pentose/glucuronate interconversions** | |  |
| **K01815** | **2.1** | **1.0** | **Metabolism** | **Carbohydrate Metabolism** | | **Pentose/glucuronate interconversions** | |  |
| **K03079** | **2.3** | **0.9** | **Metabolism** | **Carbohydrate Metabolism** | | Ascorbate and aldarate metabolism | |  |
| **K13923** | **2.6** | **1.0** | **Metabolism** | **Carbohydrate Metabolism** | | Propanoate metabolism | |  |
| **K00016** | **2.6** | **0.9** | **Metabolism** | **Carbohydrate Metabolism** | | Propanoate metabolism | |  |
| K01895 | 2.2 | 0.9 | Metabolism | Energy Metabolism | | Carbon fixation pathways in prokaryotes | |  |
| K08659 | 2.9 | 1.0 | Metabolism | Enzyme Families | | Peptidases | |  |
| K07260 | 2.5 | 1.2 | Metabolism | Enzyme Families | | Peptidases | |  |
| K01281 | 2.2 | 1.1 | Metabolism | Enzyme Families | | Peptidases | |  |
| K07778 | 2.2 | 1.1 | Metabolism | Enzyme Families | | Protein kinases | |  |
| K00712 | 2.8 | 1.3 | Metabolism | Glycan Biosynthesis and Metabolism | | Glycosyltransferases | |  |
| K12996 | 2.6 | 1.4 | Metabolism | Glycan Biosynthesis and Metabolism | | Glycosyltransferases | |  |
| K12998 | 2.5 | 1.6 | Metabolism | Glycan Biosynthesis and Metabolism | | Glycosyltransferases | |  |
| K00694 | 2.5 | 1.6 | Metabolism | Glycan Biosynthesis and Metabolism | | Glycosyltransferases | |  |
| K12997 | 2.5 | 1.3 | Metabolism | Glycan Biosynthesis and Metabolism | | Glycosyltransferases | |  |
| K00692 | 2.5 | 1.6 | Metabolism | Glycan Biosynthesis and Metabolism | | Glycosyltransferases | |  |
| K12999 | 2.4 | 1.5 | Metabolism | Glycan Biosynthesis and Metabolism | | Glycosyltransferases | |  |
| K13677 | 2.4 | 1.5 | Metabolism | Glycan Biosynthesis and Metabolism | | Glycosyltransferases | |  |
| K07272 | 2.2 | 1.4 | Metabolism | Glycan Biosynthesis and Metabolism | | Glycosyltransferases | |  |
| K01071 | 2.1 | 1.0 | Metabolism | Lipid Metabolism | | Fatty acid biosynthesis | |  |
| K00001 | 2.8 | 1.4 | Metabolism | Lipid Metabolism | | Fatty acid metabolism | |  |
| K13920 | 2.8 | 1.0 | Metabolism | Lipid Metabolism | | Glycerolipid metabolism | |  |
| K13919 | 2.7 | 1.1 | Metabolism | Lipid Metabolism | | Glycerolipid metabolism | |  |
| K01699 | 2.7 | 1.1 | Metabolism | Lipid Metabolism | | Glycerolipid metabolism | |  |
| K00005 | 2.3 | 1.1 | Metabolism | Lipid Metabolism | | Glycerolipid metabolism | |  |
| K01239 | 2.4 | 1.1 | Metabolism | Metabolism of Cofactors and Vitamins | | Nicotinate and nicotinamide metabolism | |  |
| K00259 | 2.5 | 1.6 | Metabolism | Metabolism of Other Amino Acids | | Taurine and hypotaurine metabolism | |  |
| **Cluster ASV3 (n=51, R2=34.6%, Q2=5.6%, sugar 15.7E%, sucrose 6.2E%, DeFS=4.4)** | | | | | | | |  |
| **KO** | **VIP** | **95%CI** | **Level 1** | **Level 2** | | **Level 3** | |  |
| K00818 | 2.8 | 0.4 | Metabolism | Amino Acid Metabolism | | Amino acid related enzymes | |  |
| K08969 | 3.2 | 0.6 | Metabolism | Amino Acid Metabolism | | Cysteine and methionine metabolism | |  |
| K00547 | 3.0 | 0.4 | Metabolism | Amino Acid Metabolism | | Cysteine and methionine metabolism | |  |
| K01752 | 2.6 | 0.4 | Metabolism | Amino Acid Metabolism | | Cysteine and methionine metabolism | |  |
| K05823 | 2.6 | 1.0 | Metabolism | Amino Acid Metabolism | | Lysine biosynthesis | |  |
| K04518 | 2.9 | 0.3 | Metabolism | Amino Acid Metabolism | | Phenylalanine | |  |
| K04092 | 2.7 | 0.7 | Metabolism | Amino Acid Metabolism | | Phenylalanine | |  |
| K11645 | 2.7 | 1.0 | Metabolism | Amino Acid Metabolism | | Phenylalanine | |  |
| K13853 | 2.6 | 1.4 | Metabolism | Amino Acid Metabolism | | Phenylalanine | |  |
| K09011 | 2.7 | 1.1 | Metabolism | Amino Acid Metabolism | | Valine | |  |
| K01467 | 2.6 | 0.9 | Metabolism | Biosynthesis of Other Secondary Metabolites | | beta-Lactam resistance | |  |
| **K00131** | **2.7** | **0.6** | **Metabolism** | **Carbohydrate Metabolism** | | **Glycolysis / Gluconeogenesis** | |  |
| **K01223** | **2.7** | **0.6** | **Metabolism** | **Carbohydrate Metabolism** | | **Glycolysis / Gluconeogenesis** | |  |
| **K00948** | **2.6** | **0.7** | **Metabolism** | **Carbohydrate Metabolism** | | **Pentose phosphate pathway** | |  |
| **K01839** | **2.5** | **0.5** | **Metabolism** | **Carbohydrate Metabolism** | | **Pentose phosphate pathway** | |  |
| **K00689** | **3.5** | **0.6** | **Metabolism** | **Carbohydrate Metabolism** | | **Starch and sucrose metabolism** | |  |
| **K01210** | **2.7** | **1.3** | **Metabolism** | **Carbohydrate Metabolism** | | **Starch and sucrose metabolism** | |  |
| **K01226** | **2.6** | **0.5** | **Metabolism** | **Carbohydrate Metabolism** | | **Starch and sucrose metabolism** | |  |
| **K01193** | **2.6** | **0.3** | **Metabolism** | **Carbohydrate Metabolism** | | **Starch and sucrose metabolism** | |  |
| **K00016** | **2.8** | **0.5** | **Metabolism** | **Carbohydrate Metabolism** | | Propanoate metabolism | |  |
| K02111 | 2.6 | 0.4 | Metabolism | Energy Metabolism | | Oxidative phosphorylation | |  |
| K07260 | 3.1 | 0.5 | Metabolism | Enzyme Families | | Peptidases | |  |
| K08600 | 3.1 | 0.8 | Metabolism | Enzyme Families | | Peptidases | |  |
| K08659 | 3.0 | 0.4 | Metabolism | Enzyme Families | | Peptidases | |  |
| K01281 | 2.7 | 0.8 | Metabolism | Enzyme Families | | Peptidases | |  |
| K13049 | 2.6 | 1.4 | Metabolism | Enzyme Families | | Peptidases | |  |
| K07706 | 3.0 | 0.6 | Metabolism | Enzyme Families | | Protein kinases | |  |
| K07704 | 2.7 | 1.1 | Metabolism | Enzyme Families | | Protein kinases | |  |
| K12998 | 3.5 | 0.6 | Metabolism | Glycan Biosynthesis and Metabolism | | Glycosyltransferases | |  |
| K12996 | 3.4 | 0.5 | Metabolism | Glycan Biosynthesis and Metabolism | | Glycosyltransferases | |  |
| K12999 | 3.4 | 0.8 | Metabolism | Glycan Biosynthesis and Metabolism | | Glycosyltransferases | |  |
| K00692 | 3.4 | 0.5 | Metabolism | Glycan Biosynthesis and Metabolism | | Glycosyltransferases | |  |
| K00712 | 3.3 | 0.5 | Metabolism | Glycan Biosynthesis and Metabolism | | Glycosyltransferases | |  |
| K00694 | 3.3 | 1.0 | Metabolism | Glycan Biosynthesis and Metabolism | | Glycosyltransferases | |  |
| K12997 | 3.1 | 0.3 | Metabolism | Glycan Biosynthesis and Metabolism | | Glycosyltransferases | |  |
| K13677 | 3.1 | 0.4 | Metabolism | Glycan Biosynthesis and Metabolism | | Glycosyltransferases | |  |
| K07272 | 3.0 | 0.6 | Metabolism | Glycan Biosynthesis and Metabolism | | Glycosyltransferases | |  |
| K00687 | 2.6 | 0.9 | Metabolism | Glycan Biosynthesis and Metabolism | | Peptidoglycan biosynthesis | |  |
| K12556 | 2.6 | 0.9 | Metabolism | Glycan Biosynthesis and Metabolism | | Peptidoglycan biosynthesis | |  |
| K12554 | 2.6 | 0.9 | Metabolism | Glycan Biosynthesis and Metabolism | | Peptidoglycan biosynthesis | |  |
| K05362 | 2.5 | 1.1 | Metabolism | Glycan Biosynthesis and Metabolism | | Peptidoglycan biosynthesis | |  |
| K00001 | 3.2 | 0.4 | Metabolism | Lipid Metabolism | | Fatty acid metabolism | |  |
| K00005 | 3.0 | 0.7 | Metabolism | Lipid Metabolism | | Glycerolipid metabolism | |  |
| K11754 | 2.6 | 0.6 | Metabolism | Metabolism of Cofactors and Vitamins | | Folate biosynthesis | |  |
| K01598 | 2.6 | 0.9 | Metabolism | | Metabolism of Cofactors and Vitamins | | Pantothenate and CoA biosynthesis | |
| K01922 | 2.6 | 0.9 | Metabolism | | Metabolism of Cofactors and Vitamins | | Pantothenate and CoA biosynthesis | |
| K00259 | 3.0 | 0.4 | Metabolism | | Metabolism of Other Amino Acids | | Taurine and hypotaurine metabolism | |
| K01429 | 2.5 | 0.4 | Metabolism | | Xenobiotics Biodegradation and Metabolism | | Atrazine degradation | |
| K01430 | 2.5 | 0.4 | Metabolism | | Xenobiotics Biodegradation and Metabolism | | Atrazine degradation | |
| K01428 | 2.5 | 0.4 | Metabolism | | Xenobiotics Biodegradation and Metabolism | | Atrazine degradation | |
| **Cluster ASV4 (n=8, R2=60.1%, Q2=24.6%, sugar 16.4E%, sucrose 6.2E%, DeFS=3.8)** | | | | | | | |  |
| **KO** | **VIP** | **95%CI** | **Level 1** | **Level 2** | | **Level 3** | |  |
| K00831 | 3.1 | 2.0 | Metabolism | Amino Acid Metabolism | | Amino acid related enzymes | |  |
| K08969 | 3.6 | 1.6 | Metabolism | Amino Acid Metabolism | | Cysteine and methionine metabolism | |  |
| K03918 | 2.6 | 2.4 | Metabolism | Amino Acid Metabolism | | Lysine biosynthesis | |  |
| K00215 | 2.6 | 1.6 | Metabolism | Amino Acid Metabolism | | Lysine biosynthesis | |  |
| K11645 | 3.4 | 1.1 | Metabolism | Amino Acid Metabolism | | Phenylalanine, tyrosine and tryptophan biosynthesis | |  |
| K09011 | 3.6 | 1.0 | Metabolism | Amino Acid Metabolism | | Valine, leucine and isoleucine biosynthesis | |  |
| K02352 | 3.2 | 3.0 | Metabolism | Biosynthesis of Other Secondary Metabolites | | beta-Lactam resistance | |  |
| K10775 | 3.1 | 4.1 | Metabolism | Biosynthesis of Other Secondary Metabolites | | Phenylpropanoid biosynthesis | |  |
| **K00011** | **2.7** | **5.0** | **Metabolism** | **Carbohydrate Metabolism** | | **Fructose and mannose metabolism** | |  |
| **K03332** | **2.5** | **2.7** | **Metabolism** | **Carbohydrate Metabolism** | | **Fructose and mannose metabolism** | |  |
| **K00689** | **3.9** | **1.9** | **Metabolism** | **Carbohydrate Metabolism** | | **Starch and sucrose metabolism** | |  |
| **K11195** | **2.8** | **6.2** | **Metabolism** | **Carbohydrate Metabolism** | | Amino/nucleotide sugar metabolism | |  |
| **K11194** | **2.7** | **6.2** | **Metabolism** | **Carbohydrate Metabolism** | | Amino/nucleotide sugar metabolism | |  |
| **K11196** | **2.7** | **6.2** | **Metabolism** | **Carbohydrate Metabolism** | | Amino/nucleotide sugar metabolism | |  |
| **K01840** | **2.6** | **1.2** | **Metabolism** | **Carbohydrate Metabolism** | | Amino/nucleotide sugar metabolism | |  |
| **K00024** | **2.8** | **1.7** | **Metabolism** | **Carbohydrate Metabolism** | | Glyoxylate and dicarboxylate metabolism | |  |
| **K13923** | **3.0** | **2.8** | **Metabolism** | **Carbohydrate Metabolism** | | Propanoate metabolism | |  |
| K01895 | 3.2 | 2.0 | Metabolism | Energy Metabolism | | Carbon fixation pathways in prokaryotes | |  |
| K03313 | 2.9 | 1.7 | Metabolism | Energy Metabolism | | Methane metabolism | |  |
| K02589 | 2.9 | 1.9 | Metabolism | Energy Metabolism | | Nitrogen metabolism | |  |
| K02590 | 2.7 | 1.8 | Metabolism | Energy Metabolism | | Nitrogen metabolism | |  |
| K02592 | 2.6 | 2.0 | Metabolism | Energy Metabolism | | Nitrogen metabolism | |  |
| K03885 | 2.9 | 1.0 | Metabolism | Energy Metabolism | | Oxidative phosphorylation | |  |
| K13532 | 4.4 | 7.9 | Metabolism | Enzyme Families | | Protein kinases | |  |
| K07678 | 3.4 | 2.0 | Metabolism | Enzyme Families | | Protein kinases | |  |
| K12999 | 3.8 | 1.9 | Metabolism | Glycan Biosynthesis and Metabolism | | Glycosyltransferases | |  |
| K12998 | 3.7 | 2.0 | Metabolism | Glycan Biosynthesis and Metabolism | | Glycosyltransferases | |  |
| K12997 | 3.7 | 1.5 | Metabolism | Glycan Biosynthesis and Metabolism | | Glycosyltransferases | |  |
| K03280 | 3.2 | 7.6 | Metabolism | Glycan Biosynthesis and Metabolism | | Glycosyltransferases | |  |
| K00712 | 3.2 | 2.3 | Metabolism | Glycan Biosynthesis and Metabolism | | Glycosyltransferases | |  |
| K12996 | 3.1 | 2.5 | Metabolism | Glycan Biosynthesis and Metabolism | | Glycosyltransferases | |  |
| K00694 | 3.1 | 2.3 | Metabolism | Glycan Biosynthesis and Metabolism | | Glycosyltransferases | |  |
| K00692 | 2.6 | 2.0 | Metabolism | Glycan Biosynthesis and Metabolism | | Glycosyltransferases | |  |
| K12963 | 3.8 | 2.0 | Metabolism | Glycan Biosynthesis and Metabolism | | Lipopolysaccharide biosynthesis proteins | |  |
| K00001 | 2.9 | 1.8 | Metabolism | Lipid Metabolism | | Fatty acid metabolism | |  |
| K13920 | 2.9 | 2.5 | Metabolism | Lipid Metabolism | | Glycerolipid metabolism | |  |
| K13919 | 2.7 | 2.7 | Metabolism | Lipid Metabolism | | Glycerolipid metabolism | |  |
| K01699 | 2.7 | 2.7 | Metabolism | Lipid Metabolism | | Glycerolipid metabolism | |  |
| K12441 | 3.5 | 7.0 | Metabolism | Lipid Metabolism | | Lipid biosynthesis proteins | |  |
| K01027 | 2.7 | 2.3 | Metabolism | Lipid Metabolism | | Synthesis/degradation of ketone bodies | |  |
| K01633 | 3.4 | 1.8 | Metabolism | Metabolism of Cofactors and Vitamins | | Folate biosynthesis | |  |
| K00950 | 3.2 | 2.4 | Metabolism | Metabolism of Cofactors and Vitamins | | Folate biosynthesis | |  |
| K03794 | 3.9 | 2.0 | Metabolism | Metabolism of Cofactors and Vitamins | | Porphyrin and chlorophyll metabolism | |  |
| K01719 | 3.0 | 1.9 | Metabolism | Metabolism of Cofactors and Vitamins | | Porphyrin and chlorophyll metabolism | |  |
| K09474 | 2.5 | 2.1 | Metabolism | Metabolism of Cofactors and Vitamins | | Riboflavin metabolism | |  |
| K00600 | 2.8 | 1.3 | Metabolism | Metabolism of Other Amino Acids | | Cyanoamino acid metabolism | |  |
| K02548 | 2.6 | 1.8 | Metabolism | Metabolism of Terpenoids and Polyketides | | Prenyltransferases | |  |
| K10353 | 3.4 | 6.3 | Metabolism | Nucleotide Metabolism | | Purine metabolism | |  |
| K00525 | 2.6 | 2.8 | Metabolism | Nucleotide Metabolism | | Pyrimidine metabolism | |  |
| K00531 | 4.3 | 2.5 | Metabolism | Xenobiotics Biodegradation and Metabolism | | Chloroalkane degradation | |  |
