# Supplementary material for: Oral Microbiota Profile Associates with Sugar Intake and Taste Preference Genes
Source: Nutrients. 2020 Mar 3;12(3):681. doi: 10.3390/nu12030681 (PMC7146170; doi:10.3390/nu12030681)
Supplement: Supplementary file 1 [file nutrients-12-00681-s001.zip › Table S3.docx]

| **Table S3.** Prevalence of 372 species identified from the eHOMD database at 98.5% identity and represented by at least 25 reads. Data are for cluster groups from hierarchical clustering of dichotomous variables. P-values for the prevalences are from Chi-square analyses. Species and p-values in bold are statistically significant at FDR 0.05. | | | | | |
| --- | --- | --- | --- | --- | --- |
|  | Prevalence (%) | | | | |
|  | Cluster | Cluster | Cluster | Cluster |  |
|  | H1 | H2 | H3 | H4 | p- |
| Species | n=70 | n=33 | n=48 | n=24 | value |
| *Abiotrophia defectiva* | 54,3 | 81,8 | 58,3 | 50,0 | 0,036 |
| ***Absconditabacteria (SR1) [G-1] bacterium HMT 345*** | 15,7 | 57,6 | 62,5 | 37,5 | 0,000 |
| *Absconditabacteria (SR1) [G-1] bacterium HMT 874* | 32,9 | 42,4 | 27,1 | 4,2 | 0,014 |
| ***Absconditabacteria (SR1) [G-1] bacterium HMT 875*** | 18,6 | 60,6 | 70,8 | 58,3 | 0,000 |
| *Actinomyces dentalis* | 28,6 | 54,5 | 29,2 | 37,5 | 0,055 |
| *Actinomyces georgiae* | 2,9 | 9,1 | 2,1 | 0,0 | 0,223 |
| *Actinomyces gerencseriae* | 40,0 | 42,4 | 25,0 | 50,0 | 0,150 |
| *Actinomyces graevenitzii* | 100,0 | 100,0 | 100,0 | 100,0 | 1,000 |
| *Actinomyces israelii* | 11,4 | 30,3 | 8,3 | 25,0 | 0,022 |
| *Actinomyces johnsonii* | 5,7 | 24,2 | 14,6 | 4,2 | 0,025 |
| *Actinomyces lingnae* | 98,6 | 93,9 | 95,8 | 95,8 | 0,647 |
| *Actinomyces massiliensis* | 61,4 | 84,8 | 58,3 | 62,5 | 0,068 |
| *Actinomyces naeslundii* | 88,6 | 97,0 | 89,6 | 95,8 | 0,419 |
| *Actinomyces odontolyticus* | 68,6 | 72,7 | 75,0 | 100,0 | 0,021 |
| *Actinomyces oris* | 87,1 | 90,9 | 85,4 | 79,2 | 0,637 |
| *Actinomyces sp. HMT 169* | 71,4 | 63,6 | 70,8 | 83,3 | 0,448 |
| *Actinomyces sp. HMT 170* | 24,3 | 36,4 | 16,7 | 25,0 | 0,251 |
| ***Actinomyces sp. HMT 171*** | 41,4 | 51,5 | 18,8 | 50,0 | 0,008 |
| *Actinomyces sp. HMT 172* | 90,0 | 87,9 | 93,8 | 100,0 | 0,330 |
| *Actinomyces sp. HMT 175* | 22,9 | 24,2 | 16,7 | 8,3 | 0,371 |
| ***Actinomyces sp. HMT 178*** | 38,6 | 60,6 | 18,8 | 4,2 | 0,000 |
| *Actinomyces sp. HMT 180* | 100,0 | 100,0 | 97,9 | 100,0 | 0,447 |
| *Actinomyces sp. HMT 414* | 2,9 | 12,1 | 4,2 | 4,2 | 0,242 |
| *Actinomyces sp. HMT 448* | 71,4 | 60,6 | 60,4 | 95,8 | 0,011 |
| *Actinomyces sp. HMT 525* | 10,0 | 15,2 | 10,4 | 0,0 | 0,292 |
| *Actinomyces sp. HMT 877* | 4,3 | 3,0 | 0,0 | 0,0 | 0,398 |
| *Actinomyces sp. HMT 896* | 14,3 | 27,3 | 12,5 | 12,5 | 0,269 |
| *Actinomyces sp. HMT 897* | 15,7 | 27,3 | 2,1 | 16,7 | 0,014 |
| *Actinomyces timonensis* | 1,4 | 9,1 | 2,1 | 0,0 | 0,114 |
| *Aggregatibacter actinomycetemcomitans* | 1,4 | 12,1 | 2,1 | 4,2 | 0,062 |
| *Aggregatibacter aphrophilus* | 22,9 | 27,3 | 25,0 | 4,2 | 0,151 |
| ***Aggregatibacter paraphrophilus*** | 11,4 | 42,4 | 31,3 | 29,2 | 0,004 |
| *Aggregatibacter segnis* | 58,6 | 81,8 | 64,6 | 83,3 | 0,035 |
| *Aggregatibacter sp. HMT 458* | 85,7 | 97,0 | 75,0 | 95,8 | 0,017 |
| *Aggregatibacter sp. HMT 513* | 28,6 | 39,4 | 27,1 | 33,3 | 0,640 |
| *Aggregatibacter sp. HMT 898* | 10,0 | 24,2 | 22,9 | 0,0 | 0,017 |
| ***Aggregatibacter sp. HMT 949*** | 7,1 | 27,3 | 8,3 | 4,2 | 0,009 |
| ***Alloprevotella rava*** | 55,7 | 84,8 | 75,0 | 79,2 | 0,008 |
| ***Alloprevotella sp. HMT 308*** | 95,7 | 84,8 | 70,8 | 100,0 | 0,000 |
| *Alloprevotella sp. HMT 473* | 74,3 | 87,9 | 89,6 | 83,3 | 0,135 |
| ***Alloprevotella sp. HMT 912*** | 14,3 | 48,5 | 14,6 | 20,8 | 0,001 |
| ***Alloprevotella sp. HMT 913*** | 1,4 | 27,3 | 12,5 | 4,2 | 0,000 |
| ***Alloprevotella sp. HMT 914*** | 25,7 | 78,8 | 83,3 | 58,3 | 0,000 |
| ***Alloprevotella tannerae*** | 65,7 | 90,9 | 89,6 | 91,7 | 0,001 |
| ***Alloscardovia omnicolens*** | 22,9 | 6,1 | 2,1 | 8,3 | 0,003 |
| *Atopobium parvulum* | 100,0 | 100,0 | 100,0 | 100,0 | 1,000 |
| *Atopobium rimae* | 54,3 | 72,7 | 50,0 | 62,5 | 0,189 |
| *Atopobium sp. HMT 199* | 2,9 | 12,1 | 8,3 | 4,2 | 0,281 |
| *Bacteroidaceae [G-1] bacterium HMT 272* | 0,0 | 6,1 | 2,1 | 4,2 | 0,248 |
| ***Bacteroidales [G-2] bacterium HMT 274*** | 62,9 | 97,0 | 68,8 | 100,0 | 0,000 |
| ***Bacteroidetes [G-3] bacterium HMT 280*** | 0,0 | 6,1 | 0,0 | 16,7 | 0,001 |
| ***Bacteroidetes [G-3] bacterium HMT 281*** | 0,0 | 15,2 | 0,0 | 0,0 | 0,000 |
| *Bacteroidetes [G-3] bacterium HMT 365* | 4,3 | 12,1 | 8,3 | 0,0 | 0,228 |
| *Bacteroidetes [G-3] bacterium HMT 503* | 2,9 | 9,1 | 2,1 | 8,3 | 0,324 |
| ***Bacteroidetes [G-5] bacterium HMT 505*** | 5,7 | 30,3 | 10,4 | 33,3 | 0,001 |
| *Bacteroidetes [G-5] bacterium HMT 507* | 0,0 | 6,1 | 0,0 | 8,3 | 0,932 |
| ***Bacteroidetes [G-5] bacterium HMT 511*** | 27,1 | 60,6 | 31,3 | 75,0 | 0,000 |
| *Bacteroidetes [G-7] bacterium HMT 911* | 2,9 | 3,0 | 2,1 | 0,0 | 0,860 |
| *Bergeyella sp. HMT 206* | 41,4 | 60,6 | 43,8 | 50,0 | 0,306 |
| *Bergeyella sp. HMT 322* | 94,3 | 100,0 | 97,9 | 100,0 | 0,279 |
| *Bergeyella sp. HMT 900* | 5,7 | 12,1 | 8,3 | 0,0 | 0,320 |
| ***Bergeyella sp. HMT 907*** | 8,6 | 60,6 | 8,3 | 4,2 | 0,000 |
| *Bergeyella sp. HMT 931* | 12,9 | 39,4 | 27,1 | 16,7 | 0,017 |
| *Bifidobacterium breve* | 0,0 | 0,0 | 0,0 | 4,2 | 0,097 |
| *Bifidobacterium dentium* | 11,4 | 21,2 | 6,3 | 16,7 | 0,218 |
| *Bifidobacterium longum* | 12,9 | 6,1 | 2,1 | 0,0 | 0,059 |
| ***Butyrivibrio sp. HMT 080*** | 0,0 | 18,2 | 0,0 | 4,2 | 0,000 |
| *Butyrivibrio sp. HMT 455* | 70,0 | 57,6 | 77,1 | 91,7 | 0,031 |
| *Campylobacter concisus* | 100,0 | 100,0 | 97,9 | 100,0 | 0,447 |
| *Campylobacter curvus* | 1,4 | 3,0 | 0,0 | 0,0 | 0,588 |
| *Campylobacter gracilis* | 94,3 | 100,0 | 85,4 | 91,7 | 0,088 |
| ***Campylobacter rectus*** | 68,6 | 97,0 | 91,7 | 95,8 | 0,000 |
| ***Campylobacter sp. HMT 044*** | 24,3 | 63,6 | 56,3 | 37,5 | 0,000 |
| ***Capnocytophaga gingivalis*** | 64,3 | 87,9 | 87,5 | 62,5 | 0,004 |
| *Capnocytophaga granulosa* | 62,9 | 90,9 | 58,3 | 70,8 | 0,120 |
| *Capnocytophaga haemolytica* | 15,7 | 42,4 | 14,6 | 25,0 | 0,010 |
| *Capnocytophaga leadbetteri* | 82,9 | 100,0 | 91,7 | 83,3 | 0,055 |
| ***Capnocytophaga ochracea*** | 1,4 | 15,2 | 0,0 | 4,2 | 0,003 |
| *Capnocytophaga sp. HMT 323* | 12,9 | 18,2 | 10,4 | 12,5 | 0,787 |
| *Capnocytophaga sp. HMT 324* | 11,4 | 12,1 | 4,2 | 4,2 | 0,385 |
| ***Capnocytophaga sp. HMT 326*** | 55,7 | 93,9 | 41,7 | 62,5 | 0,000 |
| ***Capnocytophaga sp. HMT 332*** | 15,7 | 57,6 | 10,4 | 4,2 | 0,000 |
| *Capnocytophaga sp. HMT 335* | 2,9 | 9,1 | 4,2 | 0,0 | 0,325 |
| *Capnocytophaga sp. HMT 336* | 38,6 | 54,5 | 31,3 | 41,7 | 0,210 |
| ***Capnocytophaga sp. HMT 338*** | 12,9 | 33,3 | 6,3 | 20,8 | 0,008 |
| *Capnocytophaga sp. HMT 380* | 7,1 | 12,1 | 6,3 | 4,2 | 0,675 |
| *Capnocytophaga sp. HMT 412* | 14,3 | 27,3 | 12,5 | 12,5 | 0,269 |
| *Capnocytophaga sp. HMT 863* | 12,9 | 24,2 | 6,3 | 8,3 | 0,101 |
| *Capnocytophaga sp. HMT 864* | 27,1 | 21,2 | 18,8 | 12,5 | 0,446 |
| *Capnocytophaga sp. HMT 901* | 1,4 | 12,1 | 4,2 | 0,0 | 0,049 |
| *Capnocytophaga sp. HMT 902* | 7,1 | 6,1 | 2,1 | 0,0 | 0,383 |
| *Capnocytophaga sp. HMT 903* | 2,9 | 18,2 | 6,3 | 8,3 | 0,050 |
| *Capnocytophaga sputigena* | 84,3 | 97,0 | 91,7 | 70,8 | 0,021 |
| *Cardiobacterium hominis* | 91,4 | 100,0 | 93,8 | 91,7 | 0,391 |
| ***Cardiobacterium valvarum*** | 44,3 | 78,8 | 58,3 | 33,3 | 0,002 |
| *Catonella morbi* | 85,7 | 87,9 | 91,7 | 95,8 | 0,507 |
| ***Catonella sp. HMT 164*** | 17,1 | 87,9 | 27,1 | 37,5 | 0,000 |
| ***Catonella sp. HMT 451*** | 0,0 | 15,2 | 0,0 | 4,2 | 0,000 |
| *Clostridiales [F-1][G-1] bacterium HMT 093* | 1,4 | 9,1 | 2,1 | 12,5 | 0,064 |
| *Corynebacterium durum* | 87,1 | 100,0 | 97,9 | 95,8 | 0,030 |
| *Corynebacterium matruchotii* | 98,6 | 100,0 | 95,8 | 100,0 | 0,433 |
| *Corynebacterium singulare* | 10,0 | 9,1 | 4,2 | 16,7 | 0,372 |
| *Cryptobacterium curtum* | 30,0 | 18,2 | 14,6 | 16,7 | 0,182 |
| *Desulfobulbus sp. HMT 041* | 0,0 | 3,0 | 0,0 | 4,2 | 0,226 |
| *Dialister invisus* | 87,1 | 93,9 | 91,7 | 100,0 | 0,245 |
| *Dialister micraerophilus* | 5,7 | 9,1 | 8,3 | 16,7 | 0,432 |
| ***Dialister pneumosintes*** | 15,7 | 45,5 | 16,7 | 41,7 | 0,001 |
| *Dialister sp. HMT 119* | 2,9 | 12,1 | 0,0 | 0,0 | 0,017 |
| *Dietzia cinnamea* | 14,3 | 24,2 | 6,3 | 8,3 | 0,106 |
| ***Eikenella corrodens*** | 34,3 | 78,8 | 41,7 | 12,5 | 0,000 |
| ***Filifactor alocis*** | 11,4 | 36,4 | 18,8 | 50,0 | 0,000 |
| *Fretibacterium fastidiosum* | 1,4 | 9,1 | 0,0 | 8,3 | 0,058 |
| *Fusobacterium gonidiaformans* | 0,0 | 6,1 | 0,0 | 4,2 | 0,085 |
| ***Fusobacterium hwasookii*** | 21,4 | 66,7 | 16,7 | 25,0 | 0,000 |
| *Fusobacterium naviforme* | 2,9 | 15,2 | 0,0 | 8,3 | 0,014 |
| *Fusobacterium necrophorum* | 7,1 | 3,0 | 6,3 | 12,5 | 0,575 |
| ***Fusobacterium nucleatum subsp. animalis*** | 82,9 | 100,0 | 64,6 | 91,7 | 0,000 |
| *Fusobacterium nucleatum subsp. nucleatum* | 2,9 | 12,1 | 6,3 | 8,3 | 0,324 |
| *Fusobacterium nucleatum subsp. polymorphum* | 85,7 | 100,0 | 87,5 | 87,5 | 0,167 |
| *Fusobacterium nucleatum subsp. vincentii* | 62,9 | 87,9 | 72,9 | 83,3 | 0,340 |
| *Fusobacterium periodonticum* | 95,7 | 100,0 | 100,0 | 100,0 | 0,205 |
| ***Fusobacterium sp. HMT 203*** | 17,1 | 48,5 | 22,9 | 16,7 | 0,004 |
| ***Fusobacterium sp. HMT 204*** | 35,7 | 78,8 | 33,3 | 50,0 | 0,000 |
| *Fusobacterium sp. HMT 248* | 22,9 | 36,4 | 33,3 | 50,0 | 0,086 |
| *Fusobacterium sp. HMT 370* | 0,0 | 6,1 | 4,2 | 12,5 | 0,050 |
| *Gemella bergeri* | 1,4 | 0,0 | 2,1 | 0,0 | 0,781 |
| *Gemella haemolysans* | 92,9 | 100,0 | 97,9 | 100,0 | 0,158 |
| *Gemella morbillorum* | 90,0 | 100,0 | 91,7 | 95,8 | 0,267 |
| *Gemella sanguinis* | 100,0 | 100,0 | 100,0 | 100,0 | 1,000 |
| *Gemella sp. HMT 928* | 0,0 | 3,0 | 0,0 | 0,0 | 0,228 |
| *Granulicatella adiacens* | 100,0 | 100,0 | 100,0 | 100,0 | 1,000 |
| ***Granulicatella elegans*** | 65,7 | 84,8 | 81,3 | 95,8 | 0,009 |
| *Haemophilus haemolyticus* | 58,6 | 84,8 | 72,9 | 83,3 | 0,017 |
| *Haemophilus influenzae* | 12,9 | 21,2 | 10,4 | 8,3 | 0,443 |
| *Haemophilus parahaemolyticus* | 48,6 | 54,5 | 52,1 | 79,2 | 0,072 |
| *Haemophilus parainfluenzae* | 100,0 | 100,0 | 100,0 | 100,0 | 1,000 |
| ***Haemophilus paraphrohaemolyticus*** | 10,0 | 18,2 | 37,5 | 54,2 | 0,000 |
| ***Haemophilus pittmaniae*** | 31,4 | 39,4 | 64,6 | 79,2 | 0,000 |
| *Haemophilus sp. HMT 036* | 77,1 | 81,8 | 79,2 | 83,3 | 0,904 |
| *Haemophilus sp. HMT 908* | 44,3 | 63,6 | 52,1 | 58,3 | 0,279 |
| ***Haemophilus sputorum*** | 48,6 | 51,5 | 72,9 | 70,8 | 0,028 |
| ***Johnsonella ignava*** | 18,6 | 60,6 | 29,2 | 25,0 | 0,000 |
| ***Johnsonella sp. HMT 166*** | 0,0 | 21,2 | 0,0 | 4,2 | 0,000 |
| ***Kingella denitrificans*** | 40,0 | 69,7 | 16,7 | 25,0 | 0,000 |
| *Kingella kingae* | 0,0 | 3,0 | 0,0 | 0,0 | 0,228 |
| *Kingella oralis* | 97,1 | 100,0 | 87,5 | 100,0 | 0,018 |
| *Kingella sp. HMT 012* | 10,0 | 15,2 | 2,1 | 0,0 | 0,059 |
| *Kingella sp. HMT 459* | 15,7 | 24,2 | 16,7 | 12,5 | 0,650 |
| *Kingella sp. HMT 932* | 2,9 | 12,1 | 4,2 | 12,5 | 0,155 |
| ***Lachnoanaerobaculum orale*** | 97,1 | 72,7 | 68,8 | 100,0 | 0,000 |
| ***Lachnoanaerobaculum saburreum*** | 14,3 | 48,5 | 8,3 | 8,3 | 0,000 |
| ***Lachnoanaerobaculum sp. HMT 083*** | 0,0 | 0,0 | 14,6 | 0,0 | 0,000 |
| *Lachnoanaerobaculum umeaense* | 92,9 | 100,0 | 100,0 | 100,0 | 0,052 |
| *Lachnospiraceae [G-2] bacterium HMT 088* | 2,9 | 9,1 | 6,3 | 4,2 | 0,578 |
| *Lachnospiraceae [G-2] bacterium HMT 096* | 32,9 | 27,3 | 35,4 | 50,0 | 0,335 |
| ***Lachnospiraceae [G-3] bacterium HMT 100*** | 40,0 | 75,8 | 62,5 | 45,8 | 0,003 |
| ***Lachnospiraceae [G-8] bacterium HMT 500*** | 0,0 | 21,2 | 20,8 | 16,7 | 0,001 |
| *Lactobacillus casei* | 10,0 | 3,0 | 2,1 | 4,2 | 0,250 |
| ***Lactobacillus crispatus*** | 0,0 | 0,0 | 0,0 | 20,8 | 0,000 |
| *Lactobacillus fermentum* | 12,9 | 9,1 | 8,3 | 20,8 | 0,441 |
| *Lactobacillus gasseri* | 8,6 | 9,1 | 2,1 | 20,8 | 0,066 |
| *Lactobacillus paracasei* | 10,0 | 6,1 | 6,3 | 12,5 | 0,741 |
| *Lactobacillus plantarum* | 4,3 | 3,0 | 6,3 | 16,7 | 0,141 |
| *Lactobacillus reuteri clade 938* | 2,9 | 9,1 | 2,1 | 0,0 | 0,223 |
| *Lactobacillus salivarius* | 4,3 | 0,0 | 0,0 | 4,2 | 0,317 |
| *Lactobacillus vaginalis* | 5,7 | 0,0 | 0,0 | 4,2 | 0,204 |
| *Lactococcus lactis* | 11,4 | 6,1 | 10,4 | 0,0 | 0,327 |
| *Lautropia mirabilis* | 95,7 | 100,0 | 91,7 | 87,5 | 0,177 |
| ***Leptotrichia buccalis*** | 41,4 | 84,8 | 31,3 | 58,3 | 0,000 |
| *Leptotrichia goodfellowii* | 55,7 | 54,5 | 56,3 | 50,0 | 0,962 |
| *Leptotrichia hofstadii* | 64,3 | 78,8 | 58,3 | 75,0 | 0,202 |
| *Leptotrichia hongkongensis* | 92,9 | 90,9 | 87,5 | 87,5 | 0,758 |
| ***Leptotrichia shahii*** | 42,9 | 66,7 | 20,8 | 25,0 | 0,000 |
| ***Leptotrichia sp. HMT 212*** | 41,4 | 72,7 | 52,1 | 29,2 | 0,005 |
| ***Leptotrichia sp. HMT 215*** | 78,6 | 100,0 | 100,0 | 95,8 | 0,000 |
| ***Leptotrichia sp. HMT 218*** | 22,9 | 30,3 | 31,3 | 62,5 | 0,005 |
| ***Leptotrichia sp. HMT 219*** | 14,3 | 42,4 | 12,5 | 0,0 | 0,000 |
| *Leptotrichia sp. HMT 221* | 78,6 | 81,8 | 72,9 | 95,8 | 0,143 |
| ***Leptotrichia sp. HMT 223*** | 14,3 | 51,5 | 12,5 | 20,8 | 0,000 |
| *Leptotrichia sp. HMT 225* | 21,4 | 39,4 | 27,1 | 8,3 | 0,049 |
| ***Leptotrichia sp. HMT 392*** | 25,7 | 78,8 | 31,3 | 0,0 | 0,000 |
| *Leptotrichia sp. HMT 417* | 94,3 | 97,0 | 91,7 | 95,8 | 0,763 |
| *Leptotrichia sp. HMT 463* | 1,4 | 6,1 | 4,2 | 12,5 | 0,156 |
| *Leptotrichia sp. HMT 498* | 31,4 | 42,4 | 14,6 | 29,2 | 0,460 |
| *Leptotrichia sp. HMT 847* | 1,4 | 3,0 | 4,2 | 4,2 | 0,810 |
| *Leptotrichia sp. HMT 879* | 2,9 | 0,0 | 0,0 | 0,0 | 0,386 |
| *Leptotrichia sp. HMT 909* | 1,4 | 9,1 | 4,2 | 4,2 | 0,329 |
| ***Leptotrichia wadei*** | 55,7 | 57,6 | 16,7 | 50,0 | 0,000 |
| ***Megasphaera micronuciformis*** | 87,1 | 51,5 | 50,0 | 79,2 | 0,000 |
| *Mogibacterium diversum* | 97,1 | 100,0 | 97,9 | 100,0 | 0,665 |
| *Mogibacterium timidum* | 1,4 | 0,0 | 0,0 | 4,2 | 0,403 |
| *Moraxella sp. HMT 276* | 0,0 | 3,0 | 0,0 | 0,0 | 0,228 |
| ***Mycoplasma faucium*** | 0,0 | 15,2 | 2,1 | 20,8 | 0,000 |
| *Mycoplasma hominis* | 1,4 | 0,0 | 0,0 | 0,0 | 0,680 |
| ***Mycoplasma orale*** | 4,3 | 18,2 | 29,2 | 8,3 | 0,001 |
| ***Mycoplasma salivarium*** | 5,7 | 39,4 | 10,4 | 20,8 | 0,000 |
| *Neisseria bacilliformis* | 25,7 | 30,3 | 16,7 | 41,7 | 0,138 |
| *Neisseria cinerea* | 25,7 | 30,3 | 29,2 | 45,8 | 0,325 |
| *Neisseria elongata* | 75,7 | 93,9 | 83,3 | 75,0 | 0,132 |
| ***Neisseria flavescens*** | 57,1 | 84,8 | 81,3 | 75,0 | 0,006 |
| *Neisseria oralis* | 37,1 | 57,6 | 29,2 | 41,7 | 0,076 |
| *Neisseria perflava* | 52,9 | 57,6 | 58,3 | 70,8 | 0,498 |
| *Neisseria sicca* | 77,1 | 84,8 | 72,9 | 79,2 | 0,648 |
| *Neisseria sp. HMT 018* | 0,0 | 0,0 | 4,2 | 4,2 | 0,225 |
| *Neisseria sp. HMT 499* | 2,9 | 9,1 | 2,1 | 4,2 | 0,403 |
| *Neisseria subflava* | 44,3 | 57,6 | 64,6 | 62,5 | 0,130 |
| ***Olsenella sp. HMT 807*** | 34,3 | 66,7 | 20,8 | 45,8 | 0,000 |
| *Oribacterium asaccharolyticum* | 95,7 | 87,9 | 89,6 | 91,7 | 0,483 |
| ***Oribacterium parvum*** | 47,1 | 81,8 | 95,8 | 75,0 | 0,000 |
| *Oribacterium sinus* | 98,6 | 97,0 | 97,9 | 100,0 | 0,842 |
| *Oribacterium sp. HMT 078* | 32,9 | 60,6 | 29,2 | 33,3 | 0,020 |
| ***Ottowia sp. HMT 894*** | 10,0 | 69,7 | 25,0 | 16,7 | 0,000 |
| *Parascardovia denticolens* | 2,9 | 6,1 | 2,1 | 8,3 | 0,525 |
| ***Parvimonas micra*** | 50,0 | 72,7 | 31,3 | 45,8 | 0,003 |
| *Parvimonas sp. HMT 110* | 12,9 | 9,1 | 8,3 | 8,3 | 0,837 |
| ***Parvimonas sp. HMT 393*** | 21,4 | 51,5 | 70,8 | 62,5 | 0,000 |
| *Peptidiphaga gingivicola* | 31,4 | 48,5 | 22,9 | 12,5 | 0,017 |
| *Peptidiphaga sp. HMT 183* | 88,6 | 93,9 | 85,4 | 91,7 | 0,647 |
| ***Peptococcus sp. HMT 167*** | 31,4 | 66,7 | 18,8 | 58,3 | 0,000 |
| ***Peptococcus sp. HMT 168*** | 41,4 | 75,8 | 100,0 | 83,3 | 0,000 |
| *Peptoniphilus lacrimalis* | 5,7 | 12,1 | 8,3 | 4,2 | 0,618 |
| *Peptostreptococcaceae [XI][G-1] sulci* | 92,9 | 97,0 | 97,9 | 100,0 | 0,342 |
| ***Peptostreptococcaceae [XI][G-2] bacterium HMT 091*** | 7,1 | 36,4 | 62,5 | 37,5 | 0,000 |
| *Peptostreptococcaceae [XI][G-4] bacterium HMT 369* | 0,0 | 9,1 | 0,0 | 8,3 | 0,014 |
| ***Peptostreptococcaceae [XI][G-5] bacterium HMT 493*** | 0,0 | 30,3 | 31,3 | 16,7 | 0,000 |
| ***Peptostreptococcaceae [XI][G-5] saphenum*** | 1,4 | 18,2 | 0,0 | 16,7 | 0,000 |
| *Peptostreptococcaceae [XI][G-6] nodatum* | 4,3 | 15,2 | 2,1 | 12,5 | 0,066 |
| ***Peptostreptococcaceae [XI][G-7] bacterium HMT 081*** | 1,4 | 24,2 | 4,2 | 8,3 | 0,000 |
| ***Peptostreptococcaceae [XI][G-7] bacterium HMT 922*** | 2,9 | 24,2 | 70,8 | 29,2 | 0,000 |
| ***Peptostreptococcaceae [XI][G-7] yurii*** | 51,4 | 72,7 | 25,0 | 41,7 | 0,004 |
| ***Peptostreptococcaceae [XI][G-9] brachy*** | 60,0 | 75,8 | 56,3 | 91,7 | 0,009 |
| ***Peptostreptococcus stomatis*** | 85,7 | 100,0 | 100,0 | 100,0 | 0,001 |
| *Porphyromonas asaccharolytica* | 1,4 | 3,0 | 2,1 | 4,2 | 0,873 |
| *Porphyromonas catoniae* | 22,9 | 54,5 | 27,1 | 29,2 | 0,011 |
| ***Porphyromonas endodontalis*** | 41,4 | 60,6 | 45,8 | 100,0 | 0,000 |
| *Porphyromonas gingivalis* | 4,3 | 6,1 | 2,1 | 8,3 | 0,650 |
| *Porphyromonas pasteri* | 91,4 | 100,0 | 95,8 | 100,0 | 0,150 |
| ***Porphyromonas sp. HMT 275*** | 32,9 | 72,7 | 37,5 | 33,3 | 0,001 |
| *Porphyromonas sp. HMT 277* | 0,0 | 3,0 | 8,3 | 12,5 | 0,036 |
| *Porphyromonas sp. HMT 278* | 37,1 | 63,6 | 37,5 | 25,0 | 0,017 |
| ***Porphyromonas sp. HMT 284*** | 42,9 | 81,8 | 60,4 | 41,7 | 0,001 |
| *Porphyromonas sp. HMT 285* | 0,0 | 9,1 | 4,2 | 0,0 | 0,053 |
| ***Porphyromonas sp. HMT 930*** | 41,4 | 78,8 | 62,5 | 58,3 | 0,003 |
| *Porphyromonas uenonis* | 8,6 | 3,0 | 12,5 | 8,3 | 0,524 |
| *Prevotella aurantiaca* | 1,4 | 6,1 | 10,4 | 0,0 | 0,083 |
| *Prevotella baroniae* | 1,4 | 9,1 | 4,2 | 8,3 | 0,269 |
| *Prevotella buccae* | 10,0 | 18,2 | 14,6 | 20,8 | 0,512 |
| ***Prevotella dentalis*** | 0,0 | 12,1 | 2,1 | 0,0 | 0,004 |
| *Prevotella denticola* | 65,7 | 60,6 | 60,4 | 91,7 | 0,042 |
| *Prevotella enoeca* | 5,7 | 15,2 | 6,3 | 8,3 | 0,393 |
| ***Prevotella fusca*** | 0,0 | 18,2 | 4,2 | 8,3 | 0,003 |
| ***Prevotella histicola*** | 100,0 | 87,9 | 81,3 | 100,0 | 0,001 |
| ***Prevotella intermedia*** | 11,4 | 36,4 | 16,7 | 45,8 | 0,001 |
| *Prevotella loescheii* | 10,0 | 27,3 | 20,8 | 4,2 | 0,036 |
| ***Prevotella maculosa*** | 41,4 | 84,8 | 50,0 | 16,7 | 0,000 |
| *Prevotella marshii* | 2,9 | 9,1 | 2,1 | 0,0 | 0,223 |
| *Prevotella melaninogenica* | 100,0 | 100,0 | 100,0 | 100,0 | 1,000 |
| ***Prevotella micans*** | 7,1 | 69,7 | 20,8 | 45,8 | 0,000 |
| *Prevotella multiformis* | 0,0 | 3,0 | 0,0 | 4,2 | 0,226 |
| *Prevotella nanceiensis* | 94,3 | 100,0 | 100,0 | 100,0 | 0,105 |
| *Prevotella nigrescens* | 88,6 | 100,0 | 83,3 | 95,8 | 0,064 |
| ***Prevotella oralis*** | 1,4 | 12,1 | 4,2 | 29,2 | 0,000 |
| *Prevotella oris* | 97,1 | 97,0 | 91,7 | 100,0 | 0,300 |
| ***Prevotella oulorum*** | 68,6 | 75,8 | 47,9 | 37,5 | 0,004 |
| *Prevotella pallens* | 97,1 | 100,0 | 97,9 | 100,0 | 0,665 |
| ***Prevotella pleuritidis*** | 27,1 | 48,5 | 29,2 | 62,5 | 0,005 |
| ***Prevotella saccharolytica*** | 28,6 | 69,7 | 33,3 | 29,2 | 0,000 |
| *Prevotella salivae* | 100,0 | 100,0 | 95,8 | 100,0 | 0,148 |
| *Prevotella scopos* | 31,4 | 51,5 | 52,1 | 66,7 | 0,011 |
| ***Prevotella shahii*** | 18,6 | 48,5 | 79,2 | 29,2 | 0,000 |
| *Prevotella sp. HMT 292* | 14,3 | 12,1 | 16,7 | 20,8 | 0,816 |
| ***Prevotella sp. HMT 300*** | 48,6 | 93,9 | 39,6 | 41,7 | 0,000 |
| ***Prevotella sp. HMT 301*** | 4,3 | 27,3 | 2,1 | 8,3 | 0,000 |
| *Prevotella sp. HMT 304* | 0,0 | 6,1 | 0,0 | 8,3 | 0,032 |
| ***Prevotella sp. HMT 305*** | 70,0 | 24,2 | 25,0 | 79,2 | 0,000 |
| *Prevotella sp. HMT 306* | 71,4 | 60,6 | 56,3 | 83,3 | 0,085 |
| *Prevotella sp. HMT 309* | 42,9 | 36,4 | 41,7 | 62,5 | 0,235 |
| *Prevotella sp. HMT 313* | 87,1 | 81,8 | 70,8 | 83,3 | 0,167 |
| *Prevotella sp. HMT 314* | 48,6 | 78,8 | 50,0 | 54,2 | 0,027 |
| ***Prevotella sp. HMT 315*** | 2,9 | 42,4 | 22,9 | 8,3 | 0,000 |
| ***Prevotella sp. HMT 317*** | 68,6 | 90,9 | 41,7 | 70,8 | 0,000 |
| *Prevotella sp. HMT 443* | 0,0 | 15,2 | 6,3 | 12,5 | 0,014 |
| ***Prevotella sp. HMT 472*** | 44,3 | 81,8 | 54,2 | 29,2 | 0,000 |
| ***Prevotella sp. HMT 475*** | 17,1 | 48,5 | 14,6 | 12,5 | 0,001 |
| ***Prevotella sp. HMT 526*** | 0,0 | 15,2 | 0,0 | 4,2 | 0,000 |
| *Prevotella sp. HMT 942* | 14,3 | 21,2 | 22,9 | 45,8 | 0,016 |
| *Prevotella veroralis* | 34,3 | 45,5 | 22,9 | 25,0 | 0,153 |
| *Rothia aeria* | 84,3 | 100,0 | 91,7 | 95,8 | 0,052 |
| *Rothia dentocariosa* | 100,0 | 100,0 | 100,0 | 100,0 | 1,000 |
| *Rothia mucilaginosa* | 100,0 | 100,0 | 97,9 | 100,0 | 0,447 |
| ***Ruminococcaceae [G-1] bacterium HMT 075*** | 61,4 | 100,0 | 87,5 | 75,0 | 0,000 |
| *Ruminococcaceae [G-2] bacterium HMT 085* | 88,6 | 97,0 | 95,8 | 87,5 | 0,279 |
| ***Saccharibacteria (TM7) [G-1] bacterium HMT 346*** | 21,4 | 42,4 | 20,8 | 4,2 | 0,007 |
| *Saccharibacteria (TM7) [G-1] bacterium HMT 347* | 15,7 | 21,2 | 8,3 | 4,2 | 0,175 |
| *Saccharibacteria (TM7) [G-1] bacterium HMT 348* | 4,3 | 12,1 | 0,0 | 0,0 | 0,034 |
| *Saccharibacteria (TM7) [G-1] bacterium HMT 349* | 8,6 | 12,1 | 4,2 | 16,7 | 0,328 |
| ***Saccharibacteria (TM7) [G-1] bacterium HMT 352*** | 78,6 | 72,7 | 95,8 | 95,8 | 0,006 |
| *Saccharibacteria (TM7) [G-1] bacterium HMT 488* | 0,0 | 3,0 | 2,1 | 0,0 | 0,475 |
| *Saccharibacteria (TM7) [G-1] bacterium HMT 869* | 1,4 | 6,1 | 4,2 | 0,0 | 0,434 |
| *Saccharibacteria (TM7) [G-1] bacterium HMT 952* | 7,1 | 18,2 | 4,2 | 0,0 | 0,042 |
| *Saccharibacteria (TM7) [G-1] bacterium HMT 957* | 0,0 | 3,0 | 0,0 | 4,2 | 0,226 |
| *Saccharibacteria (TM7) [G-2] bacterium HMT 350* | 1,4 | 6,1 | 0,0 | 0,0 | 0,173 |
| *Saccharibacteria (TM7) [G-3] bacterium HMT 351* | 40,0 | 45,5 | 54,2 | 50,0 | 0,484 |
| ***Saccharibacteria (TM7) [G-5] bacterium HMT 356*** | 12,9 | 39,4 | 4,2 | 45,8 | 0,000 |
| *Saccharibacteria (TM7) [G-6] bacterium HMT 870* | 51,4 | 63,6 | 52,1 | 41,7 | 0,422 |
| ***Scardovia wiggsiae*** | 71,4 | 48,5 | 52,1 | 83,3 | 0,008 |
| *Selenomonas artemidis* | 0,0 | 9,1 | 2,1 | 0,0 | 0,029 |
| *Selenomonas noxia* | 12,9 | 33,3 | 16,7 | 8,3 | 0,040 |
| *Selenomonas sp. HMT 136* | 32,9 | 15,2 | 27,1 | 37,5 | 0,209 |
| *Selenomonas sp. HMT 137* | 1,4 | 3,0 | 2,1 | 0,0 | 0,842 |
| *Selenomonas sp. HMT 478* | 7,1 | 0,0 | 4,2 | 0,0 | 0,243 |
| *Selenomonas sp. HMT 892* | 1,4 | 3,0 | 0,0 | 0,0 | 0,588 |
| *Selenomonas sputigena* | 2,9 | 6,1 | 2,1 | 4,2 | 0,786 |
| ***Shuttleworthia satelles*** | 21,4 | 54,5 | 35,4 | 54,2 | 0,002 |
| *Simonsiella muelleri* | 11,4 | 24,2 | 29,2 | 4,2 | 0,012 |
| *Slackia exigua* | 11,4 | 33,3 | 18,8 | 8,3 | 0,028 |
| *Sneathia amnii* | 2,9 | 0,0 | 0,0 | 4,2 | 0,422 |
| *Solobacterium moorei* | 75,7 | 60,6 | 70,8 | 62,5 | 0,375 |
| *Staphylococcus aureus* | 14,3 | 15,2 | 8,3 | 16,7 | 0,698 |
| *Staphylococcus epidermidis* | 5,7 | 6,1 | 6,3 | 0,0 | 0,676 |
| ***Stomatobaculum longum*** | 84,3 | 72,7 | 37,5 | 66,7 | 0,000 |
| ***Stomatobaculum sp. HMT 097*** | 74,3 | 90,9 | 97,9 | 95,8 | 0,001 |
| *Streptococcus anginosus* | **28,6** | **54,5** | **35,4** | **37,5** | 0,087 |
| *Streptococcus australis* | 2,9 | 18,2 | 12,5 | 12,5 | 0,069 |
| ***Streptococcus constellatus*** | 5,7 | 39,4 | 10,4 | 20,8 | 0,000 |
| *Streptococcus cristatus clade 578* | 8,6 | 12,1 | 10,4 | 4,2 | 0,756 |
| *Streptococcus gordonii* | 68,6 | 87,9 | 54,2 | 66,7 | 0,017 |
| ***Streptococcus intermedius*** | 88,6 | 90,9 | 68,8 | 87,5 | 0,015 |
| *Streptococcus lactarius* | 1,4 | 9,1 | 4,2 | 12,5 | 0,122 |
| *Streptococcus mitis* | 100,0 | 100,0 | 100,0 | 100,0 | 1,000 |
| ***Streptococcus mutans*** | 58,6 | 36,4 | 29,2 | 70,8 | 0,001 |
| *Streptococcus parasanguinis clade 411* | 100,0 | 100,0 | 91,7 | 100,0 | 0,013 |
| *Streptococcus pneumoniae* | 12,9 | 12,1 | 12,5 | 16,7 | 0,957 |
| *Streptococcus salivarius* | 100,0 | 100,0 | 100,0 | 100,0 | 1,000 |
| *Streptococcus sanguinis* | 82,9 | 100,0 | 93,8 | 87,5 | 0,040 |
| ***Streptococcus sobrinus*** | 2,9 | 0,0 | 0,0 | 33,3 | 0,000 |
| ***Streptococcus sp. HMT 056*** | 24,3 | 30,3 | 52,1 | 66,7 | 0,000 |
| *Streptococcus sp. HMT 057* | 77,1 | 72,7 | 72,9 | 100,0 | 0,044 |
| *Streptococcus sp. HMT 066* | 100,0 | 100,0 | 93,8 | 100,0 | 0,044 |
| *Streptococcus sp. HMT 074* | 94,3 | 100,0 | 97,9 | 95,8 | 0,461 |
| *Streptococcus thermophilus* | 5,7 | 6,1 | 4,2 | 0,0 | 0,672 |
| ***Tannerella forsythia*** | 14,3 | 57,6 | 10,4 | 62,5 | 0,000 |
| *Tannerella sp. HMT 286* | 74,3 | 97,0 | 83,3 | 79,2 | 0,048 |
| *Tannerella sp. HMT 808* | 14,3 | 24,2 | 16,7 | 8,3 | 0,409 |
| *Tannerella sp. HMT 916* | 0,0 | 6,1 | 2,1 | 0,0 | 0,146 |
| ***Treponema denticola*** | 8,6 | 51,5 | 22,9 | 62,5 | 0,000 |
| ***Treponema lecithinolyticum*** | 11,4 | 42,4 | 10,4 | 62,5 | 0,000 |
| ***Treponema maltophilum*** | 5,7 | 30,3 | 22,9 | 8,3 | 0,003 |
| *Treponema medium* | 2,9 | 3,0 | 0,0 | 0,0 | 0,544 |
| ***Treponema socranskii*** | 42,9 | 81,8 | 43,8 | 83,3 | 0,000 |
| ***Treponema sp. HMT 226*** | 5,7 | 27,3 | 8,3 | 4,2 | 0,004 |
| *Treponema sp. HMT 230* | 0,0 | 6,1 | 4,2 | 0,0 | 0,167 |
| *Treponema sp. HMT 231* | 40,0 | 57,6 | 37,5 | 37,5 | 0,283 |
| ***Treponema sp. HMT 234*** | 0,0 | 21,2 | 4,2 | 8,3 | 0,001 |
| *Treponema sp. HMT 235* | 0,0 | 3,0 | 0,0 | 4,2 | 0,226 |
| ***Treponema sp. HMT 237*** | 12,9 | 48,5 | 22,9 | 62,5 | 0,000 |
| *Treponema sp. HMT 238* | 1,4 | 9,1 | 0,0 | 8,3 | 0,058 |
| ***Treponema sp. HMT 246*** | 0,0 | 30,3 | 2,1 | 20,8 | 0,000 |
| *Treponema sp. HMT 251* | 2,9 | 9,1 | 0,0 | 0,0 | 0,081 |
| ***Treponema sp. HMT 253*** | 4,3 | 18,2 | 8,3 | 33,3 | 0,001 |
| *Treponema sp. HMT 256* | 0,0 | 6,1 | 0,0 | 0,0 | 0,033 |
| *Treponema sp. HMT 257* | 8,6 | 21,2 | 4,2 | 20,8 | 0,041 |
| *Treponema sp. HMT 258* | 0,0 | 6,1 | 0,0 | 0,0 | 0,033 |
| ***Treponema sp. HMT 262*** | 1,4 | 33,3 | 10,4 | 29,2 | 0,000 |
| *Treponema sp. HMT 951* | 0,0 | 3,0 | 0,0 | 0,0 | 0,228 |
| ***Treponema vincentii*** | 8,6 | 27,3 | 41,7 | 20,8 | 0,000 |
| ***Veillonella atypica*** | 95,7 | 63,6 | 60,4 | 91,7 | 0,000 |
| ***Veillonella dispar*** | 98,6 | 87,9 | 85,4 | 100,0 | 0,012 |
| *Veillonella parvula* | 54,3 | 78,8 | 75,0 | 70,8 | 0,033 |
| ***Veillonella rogosae*** | 30,0 | 78,8 | 83,3 | 62,5 | 0,000 |
| *Veillonella sp. HMT 780* | 14,3 | 33,3 | 14,6 | 25,0 | 0,091 |
| *Veillonella sp. HMT 917* | 7,1 | 6,1 | 0,0 | 12,5 | 0,156 |
