# Supplementary material for: Oral Microbiota Profile Associates with Sugar Intake and Taste Preference Genes
Source: Nutrients. 2020 Mar 3;12(3):681. doi: 10.3390/nu12030681 (PMC7146170; doi:10.3390/nu12030681)
Supplement: Supplementary file 1 [file nutrients-12-00681-s001.zip › Table S4.docx]

| **Table S4.** Bacteria and other host associated variables with a statistically significant association (PLS correlation coefficients with 95% CI that does not include 0) in PLS modelling. Cluster H3 (the cluster with lowest reported sucrose intake) was modelled against the 3 other clusters, simultaneously, or individually. Sucrose intake (E%) and caries (DeFS) are group mean values standardized for sex, age and BMI and sex and age, respectively. Variables are ordered by taxa names and other host factors at the end. Species in bold are species that commonly are referred to as acidophilic species. | |
| --- | --- |
| **Cluster H3 (n=48) vs H1, H2, H4 together (n=127)** |  |
| model R2=84%, Q2=63% | PLS |
| sucrose intake 4.9 E% | corr |
| DeFS=3.0 | coeff |
| *Absconditabacteria (SR1) [G-1] (SR1) [G-1] bacterium HMT 345* | 0.105 |
| *Absconditabacteria (SR1) [G-1] (SR1) [G-1] bacterium HMT 875* | 0.109 |
| *Aggregatibacter paraphrophilus* | 0.031 |
| *Aggregatibacter sp. HMT 898* | 0.050 |
| *Alloprevotella sp. HMT 473* | 0.042 |
| *Alloprevotella sp. HMT 914* | 0.122 |
| *Campylobacter rectus* | 0.046 |
| *Corynebacterium durum* | 0.038 |
| *Haemophilus pittmaniae* | 0.071 |
| *Lachnoanaerobaculum sp. HMT 083* | 0.120 |
| *Lachnoanaerobaculum umeaense* | 0.038 |
| *Leptotrichia sp. HMT 215* | 0.070 |
| *Mycoplasma orale* | 0.094 |
| *Oribacterium parvum* | 0.122 |
| *Parvimonas sp. HMT 393* | 0.109 |
| *Peptococcus sp. HMT 168* | 0.146 |
| *Peptostreptococcaceae [XI][G-2] [XI][G-2] bacterium HMT 091* | 0.145 |
| *Peptostreptococcaceae [XI][G-7] [XI][G-7] bacterium HMT 922* | 0.203 |
| *Prevotella shahii* | 0.165 |
| *Saccharibacteria (TM7) [G-1] (TM7) [G-1] bacterium HMT 352* | 0.069 |
| *Stomatobaculum sp. HMT 097* | 0.072 |
| *Treponema vincentii* | 0.099 |
| *Veillonella rogosae* | 0.112 |
| meatportion | 0.075 |
| blood-based foods | 0.079 |
| protein E% | 0.076 |
| Healty diet score | 0.036 |
| **Cluster H1 (n=70)** |  |
| model R2=50%, Q2=50% | PLS |
| sucrose intake=6.5 E% | corr |
| DeFS=5.8 | coeff |
| ***Actinomyces sp. HMT 171*** | 0.068 |
| ***Actinomyces sp. HMT 178*** | 0.060 |
| *Alloprevotella sp. HMT 308* | 0.099 |
| *Alloscardovia omnicolens* | 0.082 |
| ***Bifidobacterium longum*** | 0.054 |
| *Capnocytophaga sp. HMT 326* | 0.039 |
| *Capnocytophaga sp. HMT 902* | 0.032 |
| *Dietzia cinnamea* | 0.036 |
| *Lachnoanaerobaculum orale* | 0.113 |
| **lactobacilli, CFU culture** | 0.066 |
| *Leptotrichia wadei* | 0.111 |
| *Megasphaera micronuciformis* | 0.115 |
| **mutans streptococci, CFU culture** | 0.057 |
| *Olsenella sp. HMT 807* | 0.041 |
| *Peptostreptococcaceae [XI][G-7] [XI][G-7] yurii subspp. yurii & margaretiae* | 0.075 |
| *Prevotella histicola* | 0.098 |
| *Prevotella sp. HMT 305* | 0.125 |
| *Prevotella sp. HMT 306* | 0.044 |
| *Prevotella sp. HMT 313* | 0.057 |
| *Prevotella sp. HMT 317* | 0.076 |
| ***Scardovia wiggsiae*** | 0.056 |
| *Stomatobaculum longum* | 0.137 |
| *Streptococcus intermedius* | 0.070 |
| ***Streptococcus mutans*** | 0.082 |
| *Streptococcus parasanguinis clade 411* | 0.064 |
| ***Veillonella atypica*** | 0.126 |
| ***Veillonella dispar*** | 0.073 |
| sucrose, E% | 0.071 |
| sugar, E% | 0.069 |
| milk 3% | 0.023 |
| monosaccharides, E% | 0.049 |
| **Cluster H2 (n=33)** |  |
| model R2=84%, Q2=63% | PLS |
| sucrose intake 6.4 E% | corr |
| DeFS=4.5 | coeff |
| ***Actinomyces israelii*** | 0.064 |
| ***Actinomyces massiliensis*** | 0.064 |
| ***Actinomyces sp. HMT 171*** | 0.078 |
| ***Actinomyces sp. HMT 178*** | 0.097 |
| ***Actinomyces sp. HMT 897*** | 0.085 |
| *Aggregatibacter sp. HMT 458* | 0.066 |
| *Aggregatibacter sp. HMT 949* | 0.057 |
| *Alloprevotella sp. HMT 912* | 0.083 |
| *Alloprevotella sp. HMT 913* | 0.042 |
| *Atopobium rimae* | 0.051 |
| *Bacteroidales [G-2] [G-2] bacterium HMT 274* | 0.078 |
| *Bacteroidetes [G-3] [G-3] bacterium HMT 281* | 0.070 |
| *Bacteroidetes [G-5] [G-5] bacterium HMT 511* | 0.066 |
| *Bergeyella sp. HMT 206* | 0.037 |
| *Bergeyella sp. HMT 907* | 0.127 |
| ***Bifidobacterium dentium*** | 0.050 |
| *Butyrivibrio sp. HMT 080* | 0.077 |
| *Campylobacter gracilis* | 0.057 |
| *Capnocytophaga granulosa* | 0.080 |
| *Capnocytophaga haemolytica* | 0.070 |
| *Capnocytophaga ochracea* | 0.070 |
| *Capnocytophaga sp. HMT 326* | 0.120 |
| *Capnocytophaga sp. HMT 332* | 0.114 |
| *Capnocytophaga sp. HMT 338* | 0.079 |
| *Capnocytophaga sp. HMT 903* | 0.042 |
| *Cardiobacterium valvarum* | 0.048 |
| *Catonella sp. HMT 164* | 0.135 |
| *Dialister pneumosintes* | 0.071 |
| *Eikenella corrodens* | 0.083 |
| *Fusobacterium hwasookii* | 0.115 |
| *Fusobacterium naviforme* | 0.070 |
| *Fusobacterium nucleatum subsp. animalis* | 0.096 |
| *Fusobacterium nucleatum subsp. polymorphum* | 0.053 |
| *Fusobacterium sp. HMT 204* | 0.101 |
| *Gemella morbillorum* | 0.043 |
| *Haemophilus haemolyticus* | 0.032 |
| *Johnsonella sp. HMT 166* | 0.084 |
| *Kingella denitrificans* | 0.121 |
| *Kingella oralis* | 0.053 |
| *Kingella sp. HMT 012* | 0.055 |
| *Lachnoanaerobaculum saburreum* | 0.103 |
| *Leptotrichia buccalis* | 0.119 |
| *Leptotrichia shahii* | 0.104 |
| *Leptotrichia sp. HMT 219* | 0.077 |
| *Leptotrichia sp. HMT 223* | 0.096 |
| *Leptotrichia sp. HMT 392* | 0.105 |
| *Leptotrichia sp. HMT 498* | 0.070 |
| *Leptotrichia wadei* | 0.096 |
| *Mycoplasma salivarium* | 0.077 |
| *Olsenella sp. HMT 807* | 0.104 |
| *Oribacterium sp. HMT 078* | 0.070 |
| *Ottowia sp. HMT 894* | 0.100 |
| *Parvimonas micra* | 0.092 |
| *Peptococcus sp. HMT 167* | 0.109 |
| *Peptostreptococcaceae [XI][G-5] [XI][G-5] saphenum* | 0.077 |
| *Peptostreptococcaceae [XI][G-7] [XI][G-7] bacterium HMT 081* | 0.068 |
| *Peptostreptococcaceae [XI][G-7] [XI][G-7] yurii subspp. yurii & margaretiae* | 0.106 |
| *Porphyromonas catoniae* | 0.063 |
| *Porphyromonas sp. HMT 275* | 0.078 |
| *Porphyromonas sp. HMT 278* | 0.058 |
| *Prevotella fusca* | 0.052 |
| *Prevotella intermedia* | 0.051 |
| *Prevotella maculosa* | 0.080 |
| *Prevotella micans* | 0.110 |
| *Prevotella nigrescens* | 0.062 |
| *Prevotella oulorum* | 0.063 |
| *Prevotella pleuritidis* | 0.044 |
| *Prevotella saccharolytica* | 0.081 |
| *Prevotella sp. HMT 300* | 0.124 |
| *Prevotella sp. HMT 301* | 0.085 |
| *Prevotella sp. HMT 317* | 0.112 |
| *Prevotella sp. HMT 472* | 0.064 |
| *Prevotella sp. HMT 475* | 0.083 |
| *Rothia aeria* | 0.043 |
| *Saccharibacteria (TM7) [G-1] (TM7) [G-1] bacterium HMT 348* | 0.062 |
| *Saccharibacteria (TM7) [G-5] (TM7) [G-5] bacterium HMT 356* | 0.100 |
| *Selenomonas noxia* | 0.044 |
| *Stomatobaculum longum* | 0.078 |
| *Streptococcus constellatus* | 0.077 |
| *Streptococcus gordonii* | 0.080 |
| *Streptococcus intermedius* | 0.059 |
| *Streptococcus parasanguinis clade 411* | 0.043 |
| *Tannerella forsythia* | 0.114 |
| *Treponema socranskii* | 0.086 |
| *Treponema sp. HMT 237* | 0.060 |
| *Treponema sp. HMT 246* | 0.091 |
| *Treponema sp. HMT 262* | 0.064 |
| sugar, E% | 0.064 |
| sucrose, E% | 0.071 |
| **Cluster H4 (n=24)** |  |
| model R2=80%, Q2=53% | PLS |
| sucrose intake 6.0 E% | corr |
| DeFS=5.1 | coeff |
| ***Actinomyces odontolyticus*** | 0.080 |
| ***Actinomyces sp. HMT 171*** | 0.082 |
| ***Actinomyces sp. HMT 448*** | 0.095 |
| *Aggregatibacter sp. HMT 458* | 0.065 |
| *Alloprevotella sp. HMT 308* | 0.088 |
| *Bacteroidales [G-2] [G-2] bacterium HMT 274* | 0.092 |
| *Bacteroidetes [G-5] [G-5] bacterium HMT 505* | 0.071 |
| *Bacteroidetes [G-5] [G-5] bacterium HMT 511* | 0.105 |
| *Capnocytophaga sp. HMT 326* | 0.050 |
| *Capnocytophaga sp. HMT 338* | 0.055 |
| *Corynebacterium singulare* | 0.054 |
| *Dialister invisus* | 0.043 |
| *Dialister pneumosintes* | 0.069 |
| *Fusobacterium nucleatum subsp. animalis* | 0.073 |
| *Granulicatella elegans* | 0.050 |
| *Haemophilus parahaemolyticus* | 0.066 |
| *Kingella oralis* | 0.054 |
| *Lachnoanaerobaculum orale* | 0.092 |
| *Lactobacillus crispatus* | 0.098 |
| *Leptotrichia sp. HMT 221* | 0.069 |
| *Leptotrichia wadei* | 0.089 |
| *Megasphaera micronuciformis* | 0.071 |
| *Mycoplasma faucium* | 0.081 |
| *Neisseria bacilliformis* | 0.069 |
| *Olsenella sp. HMT 807* | 0.066 |
| *Peptococcus sp. HMT 167* | 0.101 |
| *Peptostreptococcaceae [XI][G-5] [XI][G-5] saphenum* | 0.087 |
| *Peptostreptococcaceae [XI][G-9] [XI][G-9] brachy* | 0.091 |
| *Porphyromonas endodontalis* | 0.135 |
| *Prevotella denticola* | 0.082 |
| *Prevotella histicola* | 0.068 |
| *Prevotella intermedia* | 0.079 |
| *Prevotella sp. HMT 305* | 0.131 |
| *Prevotella sp. HMT 306* | 0.068 |
| *Prevotella sp. HMT 317* | 0.070 |
| *Saccharibacteria (TM7) [G-5] (TM7) [G-5] bacterium HMT 356* | 0.129 |
| ***Scardovia wiggsiae*** | 0.077 |
| ***Streptococcus mutans*** | 0.101 |
| *Streptococcus parasanguinis clade 411* | 0.043 |
| ***Streptococcus sobrinus*** | 0.127 |
| *Streptococcus sp. HMT 057* | 0.084 |
| *Tannerella forsythia* | 0.139 |
| *Treponema denticola* | 0.099 |
| *Treponema lecithinolyticum* | 0.139 |
| *Treponema socranskii* | 0.096 |
| *Treponema sp. HMT 237* | 0.099 |
| ***Veillonella atypica*** | 0.082 |
| ***Veillonella dispar*** | 0.059 |
| *GNAt3* (rs11760281, AA+AG vs. GG) | 0.080 |
| milk, 1,5% | 0.065 |
| sugar, Eproc | 0.059 |
| sucrose, E% | 0.050 |
