# Supplementary material for: Oral Microbiota Profile Associates with Sugar Intake and Taste Preference Genes
Source: Nutrients. 2020 Mar 3;12(3):681. doi: 10.3390/nu12030681 (PMC7146170; doi:10.3390/nu12030681)
Supplement: Supplementary file 1 [file nutrients-12-00681-s001.zip › Table S5.docx]

| **Table S5.** Multivariate analysis (PLS-DA) was used to identify bacterial functions differentiating between cluster H3, with the lowest sucrose intake, to that of clusters H1, H2, and H4 with higher sucrose intake. Phylogenetic Investigation of Communities by Reconstruction of Unobserved States (PICRUSt 2) in QIIME 2 and Kyoto Encyclopedia of Genes and Genomes (KEGG orthology, KO) predicted bacterial functions. Functions in bold are linked to carbohydrate metabolism (e.g. starch, sucrose, and glycolysis). | | | | |
| --- | --- | --- | --- | --- |
| **Cluster-dHS1 (n=70, R2=56%, Q2=23%, sugar 15.8E%, sucrose intake=6.4 E%, DeFS=5.8)** | | | | |
| **KO** | **VIP** | **95%CI** | **Level 2** | **Level 3** |
| K01478 | 2.3 | 0.3 | Amino Acid Metabolism | Arginine and proline metabolism |
| K01243 | 2.8 | 0.5 | Amino Acid Metabolism | Cysteine and methionine metabolism |
| K00640 | 2.6 | 0.6 | Amino Acid Metabolism | Cysteine and methionine metabolism |
| K07173 | 2.5 | 0.6 | Amino Acid Metabolism | Cysteine and methionine metabolism |
| K14155 | 2.3 | 0.4 | Amino Acid Metabolism | Cysteine and methionine metabolism |
| K04518 | 2.7 | 0.4 | Amino Acid Metabolism | Phenylalanine |
| K11645 | 2.5 | 0.6 | Amino Acid Metabolism | Phenylalanine |
| K01817 | 2.4 | 0.4 | Amino Acid Metabolism | Phenylalanine |
| K09011 | 2.5 | 0.5 | Amino Acid Metabolism | Valine |
| **K00820** | **2.5** | **0.2** | **Carbohydrate Metabolism** | Amino sugar and nucleotide sugar metabolism |
| **K03079** | **2.3** | **0.8** | **Carbohydrate Metabolism** | Ascorbate and aldarate metabolism |
| **K01223** | **2.4** | **0.7** | **Carbohydrate Metabolism** | **Glycolysis / Gluconeogenesis** |
| **K00865** | **2.5** | **0.7** | **Carbohydrate Metabolism** | Glyoxylate and dicarboxylate metabolism |
| **K03780** | **2.3** | **0.8** | **Carbohydrate Metabolism** | Glyoxylate and dicarboxylate metabolism |
| **K01621** | **2.5** | **0.8** | **Carbohydrate Metabolism** | **Pentose phosphate pathway** |
| **K00874** | **2.5** | **0.4** | **Carbohydrate Metabolism** | **Pentose phosphate pathway** |
| **K01624** | **2.4** | **0.7** | **Carbohydrate Metabolism** | **Pentose phosphate pathway** |
| **K01839** | **2.3** | **0.6** | **Carbohydrate Metabolism** | **Pentose phosphate pathway** |
| **K13923** | **2.8** | **0.9** | **Carbohydrate Metabolism** | Propanoate metabolism |
| **K00016** | **2.5** | **0.5** | **Carbohydrate Metabolism** | Propanoate metabolism |
| **K01193** | **2.8** | **0.5** | **Carbohydrate Metabolism** | **Starch and sucrose metabolism** |
| **K05343** | **2.6** | **0.6** | **Carbohydrate Metabolism** | **Starch and sucrose metabolism** |
| **K01176** | **2.6** | **0.4** | **Carbohydrate Metabolism** | **Starch and sucrose metabolism** |
| **K00847** | **2.5** | **0.6** | **Carbohydrate Metabolism** | **Starch and sucrose metabolism** |
| **K01226** | **2.4** | **0.6** | **Carbohydrate Metabolism** | **Starch and sucrose metabolism** |
| **K01212** | **2.3** | **0.6** | **Carbohydrate Metabolism** | **Starch and sucrose metabolism** |
| K02114 | 2.9 | 0.4 | Energy Metabolism | Oxidative phosphorylation |
| K02111 | 2.8 | 0.5 | Energy Metabolism | Oxidative phosphorylation |
| K02115 | 2.8 | 0.5 | Energy Metabolism | Oxidative phosphorylation |
| K02110 | 2.7 | 0.6 | Energy Metabolism | Oxidative phosphorylation |
| K02109 | 2.7 | 0.6 | Energy Metabolism | Oxidative phosphorylation |
| K02112 | 2.7 | 0.6 | Energy Metabolism | Oxidative phosphorylation |
| K02113 | 2.5 | 0.5 | Energy Metabolism | Oxidative phosphorylation |
| K07386 | 2.4 | 0.3 | Enzyme Families | Peptidases |
| K01951 | 2.4 | 0.6 | Enzyme Families | Peptidases |
| K08372 | 2.3 | 0.6 | Enzyme Families | Peptidases |
| K08884 | 2.5 | 0.4 | Enzyme Families | Protein kinases |
| K13677 | 2.4 | 0.7 | Glycan Biosynthesis and Metabolism | Glycosyltransferases |
| K07272 | 2.4 | 0.6 | Glycan Biosynthesis and Metabolism | Glycosyltransferases |
| K13920 | 2.7 | 0.8 | Lipid Metabolism | Glycerolipid metabolism |
| K00005 | 2.5 | 0.6 | Lipid Metabolism | Glycerolipid metabolism |
| K13919 | 2.3 | 0.7 | Lipid Metabolism | Glycerolipid metabolism |
| K01699 | 2.3 | 0.7 | Lipid Metabolism | Glycerolipid metabolism |
| K09516 | 2.6 | 0.5 | Metabolism of Cofactors and Vitamins | Retinol metabolism |
| K11717 | 2.3 | 0.4 | Metabolism of Cofactors and Vitamins | Thiamine metabolism |
| K01911 | 2.4 | 1.0 | Metabolism of Cofactors and Vitamins | Ubiquinone and other terpenoid-quinone biosynthesis |
| K08681 | 2.6 | 0.4 | Metabolism of Cofactors and Vitamins | Vitamin B6 metabolism |
| K00868 | 2.4 | 0.4 | Metabolism of Cofactors and Vitamins | Vitamin B6 metabolism |
| K00259 | 2.4 | 0.7 | Metabolism of Other Amino Acids | Taurine and hypotaurine metabolism |
| K13787 | 2.3 | 0.6 | Metabolism of Terpenoids/Polyketides | Prenyltransferases |
| **Cluster-dHS4 (n=24, R2=44%, Q2=16%, sugar 15.6E%, sucrose intake 6.0 E%, DeFS=5.1)** | | | | |
| **KO** | **VIP** | **95%CI** | **Level 2** | **Level 3** |
| K00818 | 2.7 | 1.7 | Amino Acid Metabolism | Amino acid related enzymes |
| K01243 | 2.9 | 0.4 | Amino Acid Metabolism | Cysteine and methionine metabolism |
| K00640 | 2.6 | 1.2 | Amino Acid Metabolism | Cysteine and methionine metabolism |
| K11645 | 3.0 | 1.4 | Amino Acid Metabolism | Phenylalanine |
| K04518 | 2.9 | 0.8 | Amino Acid Metabolism | Phenylalanine |
| K13832 | 2.5 | 1.9 | Amino Acid Metabolism | Phenylalanine |
| K09011 | 3.0 | 1.2 | Amino Acid Metabolism | Valine |
| K10775 | 2.6 | 2.1 | Biosynt. of Other Secondary Metabolites | Phenylpropanoid biosynthesis |
| **K01809** | **2.6** | **0.3** | **Carbohydrate Metabolism** | Amino sugar and nucleotide sugar metabolism |
| **K00847** | **2.5** | **1.5** | **Carbohydrate Metabolism** | Amino sugar and nucleotide sugar metabolism |
| **K01223** | **2.8** | **0.9** | **Carbohydrate Metabolism** | **Glycolysis / Gluconeogenesis** |
| **K00865** | **2.6** | **0.5** | **Carbohydrate Metabolism** | **Glyoxylate and dicarboxylate metabolism** |
| **K01815** | **2.6** | **1.3** | **Carbohydrate Metabolism** | Pentose and glucuronate interconversions |
| **K01686** | **2.5** | **1.1** | **Carbohydrate Metabolism** | Pentose and glucuronate interconversions |
| **K00874** | **2.7** | **1.1** | **Carbohydrate Metabolism** | Pentose phosphate pathway |
| **K01624** | **2.5** | **1.0** | **Carbohydrate Metabolism** | Pentose phosphate pathway |
| **K13923** | **2.7** | **0.9** | **Carbohydrate Metabolism** | Propanoate metabolism |
| **K00016** | **2.6** | **0.9** | **Carbohydrate Metabolism** | Propanoate metabolism |
| **K01193** | **2.8** | **0.6** | **Carbohydrate Metabolism** | **Starch and sucrose metabolism** |
| **K01176** | **2.8** | **0.8** | **Carbohydrate Metabolism** | **Starch and sucrose metabolism** |
| **K01226** | **2.6** | **1.3** | **Carbohydrate Metabolism** | **Starch and sucrose metabolism** |
| **K01212** | **2.6** | **1.9** | **Carbohydrate Metabolism** | **Starch and sucrose metabolism** |
| **K00689** | **2.4** | **2.0** | **Carbohydrate Metabolism** | **Starch and sucrose metabolism** |
| K01895 | 2.5 | 1.4 | Energy Metabolism | Carbon fixation pathways in prokaryotes |
| K02111 | 2.9 | 1.4 | Energy Metabolism | Oxidative phosphorylation |
| K02115 | 2.7 | 1.3 | Energy Metabolism | Oxidative phosphorylation |
| K02110 | 2.7 | 1.3 | Energy Metabolism | Oxidative phosphorylation |
| K02113 | 2.7 | 1.2 | Energy Metabolism | Oxidative phosphorylation |
| K02112 | 2.7 | 1.3 | Energy Metabolism | Oxidative phosphorylation |
| K02114 | 2.7 | 1.3 | Energy Metabolism | Oxidative phosphorylation |
| K02109 | 2.6 | 1.4 | Energy Metabolism | Oxidative phosphorylation |
| K07386 | 2.5 | 0.9 | Enzyme Families | Peptidases |
| K07260 | 2.5 | 1.4 | Enzyme Families | Peptidases |
| K08884 | 2.5 | 0.8 | Enzyme Families | Protein kinases |
| K12996 | 2.7 | 2.0 | Glycan Biosynthesis and Metabolism | Glycosyltransferases |
| K13677 | 2.7 | 1.8 | Glycan Biosynthesis and Metabolism | Glycosyltransferases |
| K00712 | 2.7 | 1.6 | Glycan Biosynthesis and Metabolism | Glycosyltransferases |
| K12998 | 2.6 | 1.9 | Glycan Biosynthesis and Metabolism | Glycosyltransferases |
| K00692 | 2.6 | 1.8 | Glycan Biosynthesis and Metabolism | Glycosyltransferases |
| K07272 | 2.6 | 1.7 | Glycan Biosynthesis and Metabolism | Glycosyltransferases |
| K00005 | 2.8 | 1.6 | Lipid Metabolism | Glycerolipid metabolism |
| K03426 | 2.5 | 1.2 | Metabolism of Cofactors and Vitamins | Nicotinate and nicotinamide metabolism |
| K09516 | 2.7 | 1.9 | Metabolism of Cofactors and Vitamins | Retinol metabolism |
| K01911 | 2.7 | 1.3 | Metabolism of Cofactors and Vitamins | Ubiquinone and other terpenoid-quinone biosynthesis |
| K02551 | 2.5 | 1.1 | Metabolism of Cofactors and Vitamins | Ubiquinone and other terpenoid-quinone biosynthesis |
| K01661 | 2.5 | 1.1 | Metabolism of Cofactors and Vitamins | Ubiquinone and other terpenoid-quinone biosynthesis |
| K08681 | 2.8 | 0.5 | Metabolism of Cofactors and Vitamins | Vitamin B6 metabolism |
| K00868 | 2.6 | 1.3 | Metabolism of Cofactors and Vitamins | Vitamin B6 metabolism |
| K06215 | 2.5 | 0.7 | Metabolism of Cofactors and Vitamins | Vitamin B6 metabolism |
| K00259 | 2.5 | 1.5 | Metabolism of Other Amino Acids | Taurine and hypotaurine metabolism |
| **Cluster-dHS2 (n=33, R2=39%m, Q2=6%, sugar 15.9E%, sucrose intake 6.5 E%, DeFS=4.5)** | | | | |
| **KO** | **VIP** | **95%CI** | **Level 2** | **Level 3** |
| **K00700** | **2.1** | **1.2** | **Carbohydrate Metabolism** | **Starch and sucrose metabolism** |
| K01770 | 2.3 | 0.7 | Metabolism of Terpenoids/Polyketides | Terpenoid backbone biosynthesis |
| K00099 | 2.1 | 0.6 | Metabolism of Terpenoids/Polyketides | Terpenoid backbone biosynthesis |
| K03526 | 2.0 | 0.6 | Metabolism of Terpenoids/Polyketides | Terpenoid backbone biosynthesis |
| K00991 | 2.0 | 0.7 | Metabolism of Terpenoids/Polyketides | Terpenoid backbone biosynthesis |
| K00788 | 2.0 | 1.0 | Metabolism of Cofactors and Vitamins | Thiamine metabolism |
